# Impact of C‐terminal truncations in the *Arabidopsis* Rab escort protein (REP) on REP–Rab interaction and plant fertility

**DOI:** 10.1111/tpj.15519

**Published:** 2021-10-16

**Authors:** Małgorzata Gutkowska, Magdalena Kaus‐Drobek, Marta Hoffman‐Sommer, Magdalena Małgorzata Pamuła, Anna Daria Leja, Małgorzata Perycz, Małgorzata Lichocka, Agnieszka Witek, Magdalena Wojtas, Michał Dadlez, Ewa Swiezewska, Liliana Surmacz

**Affiliations:** ^1^ Institute of Biochemistry and Biophysics Polish Academy of Sciences ul. Pawinskiego 5a, 02‐106 Warsaw Poland; ^2^ Mossakowski Medical Research Centre Polish Academy of Sciences ul. Pawinskiego 5, 02‐106 Warsaw Poland; ^3^ Institute of Computer Science Polish Academy of Sciences ul. Jana Kazimierza 5 01‐248 Warsaw Poland

**Keywords:** Rab escort protein, protein geranylgeranylation, *Arabidopsis thaliana*, Rab proteins, Pollen, HDX‐MS

## Abstract

Lipid anchors are common post‐translational modifications for proteins engaged in signaling and vesicular transport in eukaryotic cells. Rab proteins are geranylgeranylated at their C‐termini, a modification which is important for their stable binding to lipid bilayers. The Rab escort protein (REP) is an accessory protein of the Rab geranylgeranyl transferase (RGT) complex and it is obligatory for Rab prenylation. While REP–Rab interactions have been studied by biochemical, structural, and genetic methods in animals and yeast, data on the plant RGT complex are still limited. Here we use hydrogen–deuterium exchange mass spectrometry (HDX‐MS) to describe the structural basis of plant REP–Rab binding. The obtained results show that the interaction of REP with Rabs is highly dynamic and involves specific structural changes in both partners. In some cases the Rab and REP regions involved in the interaction are molecule‐specific, and in other cases they are common for a subset of Rabs. In particular, the C‐terminus of REP is not involved in binding of unprenylated Rab proteins in plants, in contrast to mammalian REP. In line with this, a C‐terminal REP truncation does not have pronounced phenotypic effects *in planta*. On the contrary, a complete lack of functional REP leads to male sterility in Arabidopsis: pollen grains develop in the anthers, but they do not germinate efficiently and hence are unable to transmit the mutated allele. The presented data show that the mechanism of action of REP in the process of Rab geranylgeranylation is different in plants than in animals or yeast.

## Significance Statement

The Rab escort protein (REP) is part of the Rab geranylgeranyl transferase (RGT) complex and it is obligatory for Rab prenylation. We show by biochemical, biophysical, and genetic methods that the mechanism of action of REP, in particular the role of its C‐terminus in Rab binding and prenylation, is different in plants than in animals or yeast.

## INTRODUCTION

Rab proteins belong to a large family of small GTPases – regulatory proteins important for vesicular transport and signal transduction in the cell (Pfeffer, 2017). In all eukaryotes the intracellular trafficking of membranes, proteins, and polysaccharides is dependent on Rab functioning. In plants a very broad expansion of the Rab family is observed (57 genes), which is comparable to that observed in mammals (60 genes), while the basic functions of Rabs in intracellular traffic are fulfilled by a mere 11 genes present in yeast (Rutherford and Moore, 2002). Specific Rab subclasses have undergone expansion in plants (e.g., the Rab‐A family is expanded compared to Rab11 in mammals), while other subclasses are more expanded in mammals than in plants (e.g., the small Rab‐E group in plants compared to the broad expansion of Rab8‐related groups in mammals; Rutherford and Moore, 2002). This is likely connected to the fact that plants, being sessile organisms, have many additional requirements for specific functions that are absent or less developed in motile organisms. These functions may include the biosynthesis and modification of cell walls, defense against pathogens, phytohormone transport, or maintaining ion homeostasis and regulating vacuolar storage (Elliott et al., 2020; Nielsen, 2020; Rivero et al., 2019). The need for both constitutive and specialized traffic puts Rab proteins in a central position of plant growth, development, and reproduction. Plant *rab* mutants, carrying defects either in a single or in multiple (redundant) Rab‐encoding genes, are often pollen sterile or show pleiotropic phenotypes in the sporophyte (reviewed in Elliott et al., 2020; Nielsen, 2020; Pfeffer, 2017; Rivero et al., 2019).

All Rab proteins bind GTP and hydrolyze it to GDP. The conformation of a Rab protein, and hence its ability to interact with other proteins, is strictly dependent on the nature of the nucleotide bound (Pfeffer, 2017). GTP‐bound Rabs are able to bind and modulate the activity of a wide range of effector proteins: motor proteins, phosphoinosite‐synthesizing enzymes, ubiquitin hydrolases, subunits of tethering and membrane fusion complexes, and many more (Pylypenko et al., 2018). For this reason, the GTP‐bound form is considered the active form of the protein. The intrinsic rate of GTP hydrolysis is slow, but it is accelerated by GTPase‐activating proteins (GAPs), which are specific for various Rab subfamilies. GDP‐bound Rabs have much lower affinity for effector proteins and are referred to as inactive. Activation of Rab proteins requires exchange of the bound GDP for a fresh GTP molecule – a process regulated by members of the diverse and evolutionarily unrelated to each other group of guanine–nucleotide exchange factors (GEFs) (Pylypenko et al., 2018).

In Rab functioning, this GTPase cycle is accompanied by a membrane insertion–extraction cycle, which is subject to separate regulatory mechanisms, other than the GAP versus GEF activity (see Kalde et al., 2019 and references therein). Since Rab effectors are membrane‐bound proteins, Rabs also need to localize to membranes. For the majority of Rabs this is achieved by adding two geranylgeranyl anchors to cysteine residues near the Rab C‐terminus (Leung et al., 2007; Shinde and Maddika, 2018). This is a very stable modification which prevents spontaneous dissociation of the Rab molecule from the membrane (Shahinian and Silvius, 1995), but at the same time the protein remains more prone to extraction and recycling from the lipid bilayer than a transmembrane protein. Membrane extraction is catalyzed by a guanine–nucleotide dissociation inhibitor (GDI), which remains bound to the extracted, prenylated Rab‐GDP molecule, but can be removed by a GDI displacement factor (GDF), which in this way unmasks the lipid anchor and allows membrane re‐insertion (Nielsen, 2020; Pfeffer, 2017). Lately it became apparent that apart from its anchoring role, the geranylgeranyl moiety itself may also form part of a recognition signal between the Rab and its effectors (Lee et al., 2020). Maintaining the equilibrium of membrane‐bound versus cytoplasmic and GTP‐activated versus GDP‐inactivated Rabs enables cells to fine‐tune the regulation of Rab activity (Bezeljak et al., 2020).

The post‐translational geranylgeranylation of Rab GTPases is always catalyzed by the Rab geranylgeranyl transferase (RGT) complex, composed of three subunits: two catalytic geranylgeranyl transferase subunits (RGTA and RGTB; Guo et al., 2008) and the substrate‐presenting subunit Rab escort protein (REP), which is an obligatory component of the complex. REP, through its Rab‐binding platform (RBP), is crucial for Rab recognition by the RGT complex (reviewed in Gutkowska and Swiezewska, 2012).

Mammalian REP is composed of two subdomains followed by a long C‐terminal tail. It shares structural similarities with Rab GDI, consistent with the preference of both proteins for the GDP‐bound forms of Rabs (Alexandrov et al., 1994; Alory and Balch, 2003; Seabra, 1996). Crystal structures of the mammalian Rab–REP complex, as well as biochemical data, suggest high importance of the REP C‐terminal region in the immobilization and positioning of the hypervariable Rab C‐terminus, which needs to be correctly presented for prenylation by the RGT complex (Pylypenko et al., 2003; Rak et al., 2004; Zhang et al., 2000). In both REP and GDI structures, the electron density for the C‐terminal tail is very low, suggesting that this element does not localize inside the protein structure (Pylypenko et al., 2003; Rak et al., 2004; Schalk et al., 1996). The specificity of Rab prenylation on the two adjacent C‐terminal cysteines is exclusively dependent on the REP–Rab interaction, and not on the peptide motif surrounding the cysteines (Anant et al., 1998; Shi et al., 2016).

In yeast the REP–Rab interaction was studied by genetic (Benito‐Moreno et al., 1994; Miaczynska et al., 1997; Ragnini et al., 1994) and biochemical (Dursina et al., 2002; Jiang and Ferro‐Novick, 1994) methods. Interestingly, the available biochemical data on mammalian and yeast REP are not fully convergent. The binding affinities for Rabs (Dursina et al., 2002; Pylypenko et al., 2006; Thoma et al., 2001), the prenylation consensus sequences, and the hierarchy of prenylation of Rabs (Bialek‐Wyrzykowska et al., 2000; Kohnke et al., 2013; Storck et al., 2019) differ between the kingdoms of life.

Several described *REP* mutants provide insights into REP function. In the yeast *Saccharomyces cerevisiae*, a complete lack of REP (Mrs6p) activity is lethal (Jiang and Ferro‐Novick, 1994), while a conditional mutation disrupts membrane association of Rabs and intracellular trafficking (Fujimura et al., 1994). However, genetic and biochemical data show that C‐terminal truncations of Mrs6p influence viability and fertility only mildly (Bauer et al., 1996; Bialek‐Wyrzykowska et al., 2000; Miaczynska et al., 1997). Also for vertebrates a complete lack of functional REP is lethal, as has been shown for the fish *Danio rerio* (Moosajee et al., 2009) and for mice (Shi et al., 2004). In humans two REP homologues have been identified and while the consequences of *REP‐2 (CHML)* deficiency are not known (Cremers et al., 1994), the deficiency of *REP‐1 (CHM)* leads to progressive retinal dystrophy – choroideremia (Andres et al., 1993).

For plants much less is known about REP. The lack of functional REP has been studied only in the moss *Physcomitrella patens*, where it is lethal (Thole et al., 2014). In Arabidopsis a single gene encoding REP has been identified (Hala et al., 2005), and the encoded protein and its cellular function have been characterized (Hala et al., 2005; Shi et al., 2016; Wojtas et al., 2007). Taking into account all the processes that plant Rab proteins are engaged in (reviewed in Elliott et al., 2020; Minamino and Ueda, 2019; Rivero et al., 2019, and others), it might be expected that a *REP* knock‐out in Arabidopsis would cause sterility, similarly to a complete loss of *RGTB* function (Gutkowska et al., 2015). However, the effects of a partial reduction of REP activity are difficult to foresee, even taking into account that partial loss of RGTB activity has already been described (Hala et al., 2010; Rojek et al., 2021a,b). Also, no structural data for plant REP are available, neither alone nor in a REP–Rab complex.

For these reasons, in this work we decided to study plant REP–Rab interactions by combining structural, genetic, and cell biology methods. First we aimed to characterize the REP–Rab interaction in *Arabidopsis thaliana* by structural methods, using hydrogen–deuterium (H‐D) exchange mass spectrometry (HDX‐MS). This method allows the examination of protein–protein interactions in solution, in native conditions, and therefore may be treated as complementary to crystallography (Zheng et al., 2019). In HDX‐MS no artificial, crystallization‐forced constraints are imposed onto the protein and even relatively unstructured and highly mobile elements may be studied (Hodge et al., 2020). In particular we wanted to learn more about plant‐specific, unconserved regions of the REP protein, especially the C‐terminal tail, predicted to be mobile and highly unstructured (Rasteiro and Pereira‐Leal, 2007). HDX‐MS was, therefore, the method of choice. In parallel we studied the *in planta* consequences of C‐terminal REP truncations in *A. thaliana*.

## RESULTS

### Recombinant plant REP forms *in vitro* stoichiometric complexes with selected Rab proteins

We were interested to find out which parts of the REP and Rab proteins are engaged in complex formation in plants. In particular, we wanted to know if the REP C‐terminal tail takes part in the interaction and whether the same regions of REP were involved with different Rabs. At the same time, we wanted to analyze possible structural changes occurring in the Rab proteins upon REP binding. To answer these questions, we decided to apply HDX‐MS. This method relies on the naturally occurring proton exchange that takes place in solution, especially for amide protons. If heavy water (D_2_O) is used instead of H_2_O, proton exchange can be monitored by MS. Protein regions localized in more exposed areas exchange amide protons with higher efficiency, while more hidden regions, for example those that form the protein hydrophobic core, exchange protons at a slower rate. Comparison of proton exchange rates between sample and control enables identification of peptides that undergo structural changes upon binding of the partner protein (Zheng et al., 2019). The experiment can be performed as a time course, yielding additional temporal information.

**Figure 1:**
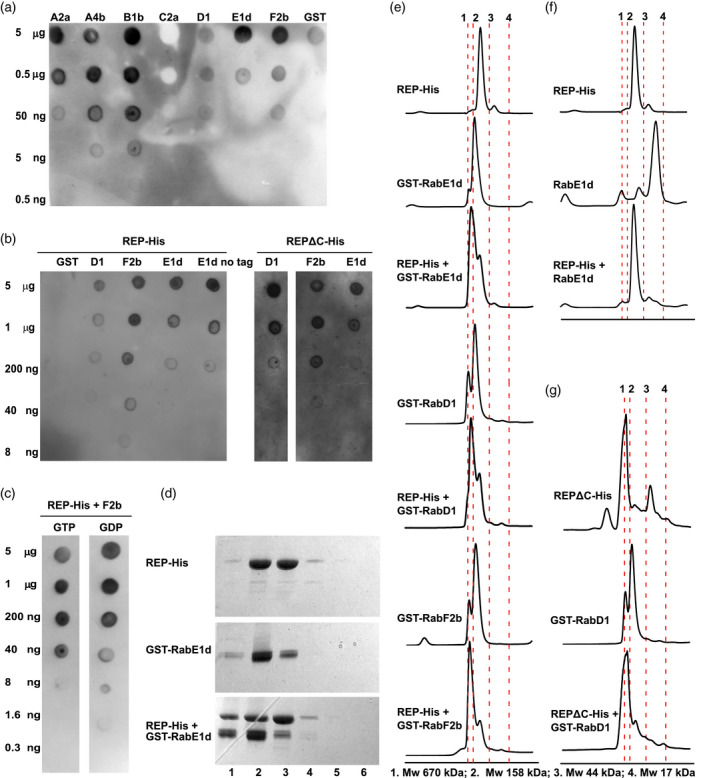
Interaction of the REP protein and its C‐terminally truncated mutant with Rab proteins *in vitro*. (a) REP‐His binds to Rab proteins with different affinities. Purified GST‐Rab proteins were spotted on nitrocellulose membrane in serial dilutions as denoted and overlayed with a solution of purified REP‐His. REP binding was revealed with an anti‐His antibody. (b) REP‐His protein and its C‐terminally truncated variant REPΔC‐His interact with selected GST‐Rab proteins equally well, but do not interact with the GST tag. *E1d no tag* represents purified Rab‐E1d cleaved from its GST tag. (c) Plant REP–Rab binding is mildly influenced by guanine nucleotide identity. GTP or GDP was pre‐incubated with the GST‐Rab protein before membrane spotting and added to the Rab–REP binding solution. Panels (a–c) show representative blot overlay assay results. Purity of proteins is shown in Figure S1(a) and anti‐His antibody specificity is shown in Figure S1(b). (d) Representative result of the SEC experiment on REP–Rab complex formation in solution. REP‐His and GST‐Rab‐E1d were pre‐incubated in the presence of GDP and resolved on a SEC column. One‐milliliter fractions of eluate were gathered and resolved by SDS‐PAGE. Numbers below the panels mark the fraction of eluate starting from void volume of the column. Note the presence of the REP–Rab complex in fraction 1. The respective chromatogram is shown in panel (e). (e) Selected GST‐Rab proteins form complexes with the REP‐His protein in solution in the presence of GDP. (f) Rab‐E1d cleaved from its GST tag forms complexes with REP‐His equally well as the tagged protein; compare with panel (e). (g) REPΔC‐His binds GST‐Rab proteins in solution. Note the high‐molecular‐mass species in REPΔC‐His, representing probably oligomers/aggregates. Panels (e–g) show representative SEC chromatograms. Vertical dashed lines mark the retention times of molecular weight standards as denoted under the graphs. Respective SDS‐PAGE gels are shown in Figure S1(c–e).

The affinity of Rabs towards the REP protein was previously addressed in several studies in the yeast model (Dursina et al., 2002; Pylypenko et al., 2006), and later also in mammalian cells (Kohnke et al., 2013; Storck et al., 2019). In order to choose Rab proteins for our structural studies, we first purified recombinant REP and seven different Rabs (Figure S1a) and then performed *in vitro* protein–protein binding experiments by means of a protein overlay assay (Figure 1a). The anti‐His antibody used for the overlay assays did not show any cross‐reactivity with the purified GST‐Rabs (Figure S1b). Recombinant REP‐His and selected recombinant GST‐Rab proteins formed complexes with different affinities, with differences reaching two orders of magnitude (Figure 1a). Binding of REP‐His to GST alone was negligible (Figure 1a,b), and removal of the GST tag from Rab‐E1d had no influence on the interaction (Figure 1b), showing that it was the Rab itself that bound REP. To see whether the binding was dependent on the REP C‐terminus, we purified a truncated form of the protein, lacking 30 C‐terminal amino acids, which we called REPΔC‐His. The truncated mutant bound the tested GST‐Rabs equally well as wild‐type (WT) REP‐His (Figure 1b). Binding of REP‐His to GST‐Rabs was slightly stronger in the presence of GDP than in the presence of GTP (Figure 1c).

Based on the availability of crystal structures, we chose GDP‐bound forms of Rab‐F2b, Rab‐E1d, and Rab‐D1 as examples of three divergent Rabs for further structural elucidations (Uejima et al., 2010 present the structure of Rab‐F2b; Cai et al., 2008 present the structure of yeast Ypt1, a close homologue of Rab‐D1, and Itzen et al., 2006 present the structure of Rab8, a human homologue of Rab‐E1d). Next we performed size‐exclusion chromatography (SEC) of REP‐His/GST‐Rab‐F2b, REP‐His/GST‐Rab‐D1, and REP‐His/GST‐Rab‐E1d complexes formed in the presence of GDP (Figure 1d,e and Figure S1c) and gathered the fractions of (presumably) stoichiometric complexes. These fractions were then concentrated and used for further experiments. To make sure that it is the Rab itself, and not the GST, that interacts with REP‐His, we incubated Rab‐E1d cleaved from the GST tag with REP‐His, and here we observed a clear shift for Rab‐E1d (Figure 1f and Figure S1d). As an additional control we also incubated REP‐His with GST alone and here we did not find any binding (Figure S1d).

To confirm the dot‐blot results for REPΔC‐His, we performed the SEC experiment for the truncated REP version. Recombinant REPΔC‐His protein showed a higher tendency to oligomerize/aggregate upon prolonged incubation at room temperature than WT REP (Figure S1e). Despite this, when we incubated REPΔC‐His with GST‐Rab‐E1d or GST‐Rab‐D1, the binding remained detectable (Figure 1g and Figure S1e,f). This suggested that the REP C‐terminus is not indispensable for the REP–Rab interaction.

### HDX‐MS enables us to study the formation of REP complexes with Rab proteins in solution

**Figure 2:**
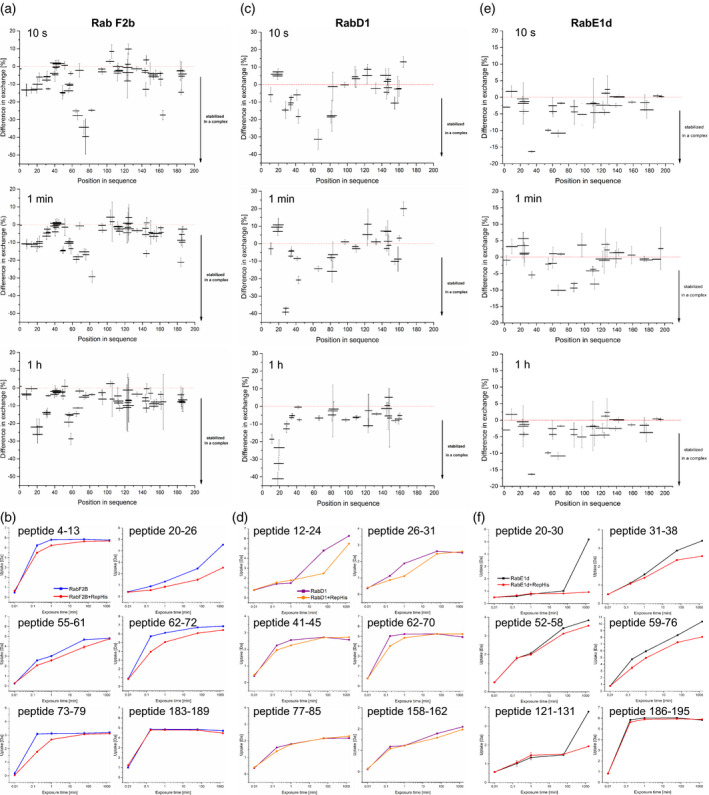
HDX‐MS analysis of Rab structural changes upon REP binding. (a, c, e) Relative difference in H‐D exchange for Rab peptides upon REP binding at 10 sec, 1 min, and 1 h [%]. Horizontal bars represent individual peptides distributed along the protein amino acid sequence, vertical bars represent SD from the mean relative difference in H‐D exchange for a given peptide measured in triplicate. Minimal exchange was measured for a peptide not incubated with D_2_O, maximal exchange was measured after 48 h of incubation. Results were recorded for (a) GST‐Rab‐F2b, (c) GST‐Rab‐D1, and (e) GST‐Rab‐E1d upon REP‐His binding in the presence of GDP. (b, d, f) Deuterium uptake by selected Rab peptides upon REP binding in a time course of 1 sec to 48 h [Da]. Each point represents the mean centroid mass of a peptide coming from protein or complex incubated in D_2_O ± SD. Amino acid numbers in Rab peptide sequences are denoted on the graphs. Corresponding Rab amino acid sequences are shown in Figure 4(c).

**Figure 3:**
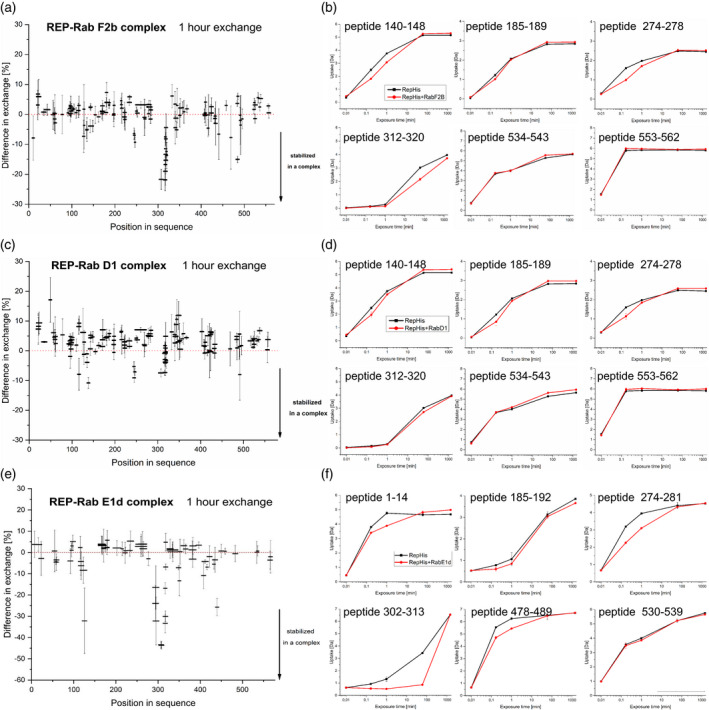
HDX‐MS analysis of REP upon binding of Rab proteins. (a, c, e) Relative difference in H‐D exchange for REP peptides upon binding of Rab proteins after 1 h incubation in D_2_O [%]. Horizontal bars represent individual peptides distributed along the protein amino acid sequence, vertical bars represent SD from the mean relative difference in H‐D exchange for a given peptide measured in triplicate. Minimal exchange was measured for a peptide not incubated with D_2_O (1 sec time point), maximal exchange was measured after 48 h of incubation. Results were recorded for REP‐His binding to (a) GST‐Rab‐F2b, (c) GST‐Rab‐D1, and (e) GST‐Rab‐E1d in the presence of GDP. (b, d, f) Deuterium uptake by selected REP peptides upon Rab binding in a time course of 1 sec to 48 h [Da]. Each point represents the mean centroid mass of a peptide coming from protein or complex incubated in D_2_O ± SD. Amino acid numbers in REP peptide sequences are denoted on the graphs. Results were recorded for REP‐His binding in the presence of GDP to (b) GST‐Rab‐F2b, (d) GST‐Rab‐D1, and (f) GST‐Rab‐E1d.

First, we experimentally defined peptide coverage of the studied proteins upon pepsin cleavage and LC‐electron spray ionization (ESI)‐MS/MS peptide fragmentation and sequencing (Figure S2). This allowed us to construct a peptide library which then served as a peptide reference list for the interaction partners. Next, we incubated each protein alone (apo form) in deuterium‐containing buffer for 10 sec, 1 min, and 1 h in triplicate and we monitored the levels of H‐D exchange for each peptide from the reference list. Two control experiments for minimal and maximal exchange were performed. In parallel, we analyzed the H‐D exchange levels in stoichiometric complexes of REP‐His with GST‐Rab‐F2b, GST‐Rab‐D1, or GST‐Rab‐E1d which have been obtained from SEC experiments. The results are shown in Figures 2 and 3.

**Figure 4:**
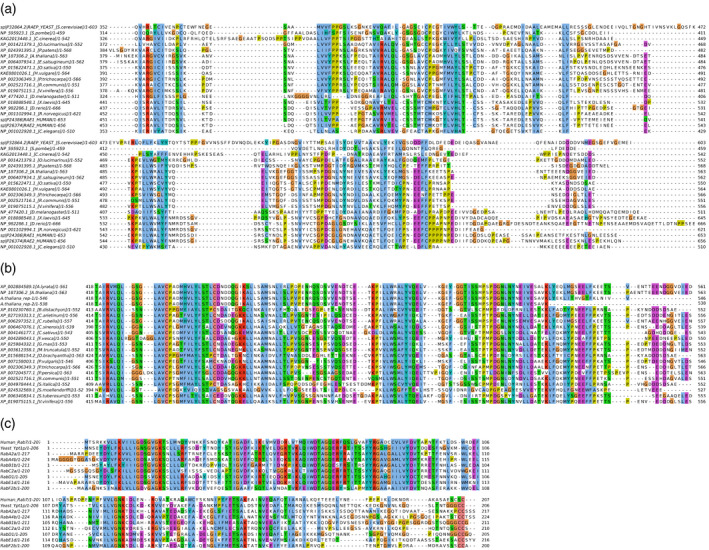
Alignments of Rab and REP sequences. (a) Alignment of the variable C‐terminus of the REP protein (or putative REP) from organisms representing different phylogenetic groups. Note the low conservation of this fragment. Alignment of full‐length REP amino acid sequences is shown in Figure S4. (b) Alignment of the *Arabidopsis thaliana* REP C‐terminus with corresponding C‐terminal fragments from other plant species. Note the relatively good conservation of this fragment. *rep‐1* and *rep‐2* represent the proteins present in Arabidopsis *rep‐1* and *rep‐2* mutant lines, respectively. (c) Alignment of Arabidopsis Rab protein sequences used in this study with the human Rab7 sequence, for which the crystal structure was solved (in complex with rat REP). Note the low conservation in the C‐terminal region of Rab sequences.

Then we conducted a bioinformatical analysis of the assayed proteins, in order to be able to map REP and Rab peptides that undergo increased protection or exposure upon complex formation. We aligned REP amino acid sequences from different organisms (Figure S4; subalignment of C‐terminal tail regions is shown in Figure 4a). The alignment clearly shows the low conservation of the C‐terminal tail. However, when only plant species are considered, the C‐terminal tail shows significantly higher conservation (Figure 4b), suggesting that its role might be different in various groups of organisms. We additionally analyzed secondary structure predictions for *A. thaliana* REP (Figure S5), which proved consistent with the idea of the C‐terminus being disordered. We also aligned selected *A. thaliana* Rab sequences with human Rab7 (Figure 4c).

To complement the alignments, we then created approximate structural models of Arabidopsis REP, Rab‐F2b, Rab‐D1, and Rab‐E1d (Figure S6). We used the PHYRE^2^ server, based on a hidden Markov model calculation method (Kelley et al., 2015). The REP model is based on the crystal structures of the mammalian REP–Rab and GDI–Rab complexes, while Rab models are based on known crystal structures of Rab proteins. All these resources allowed us to map changes that complex formation elicits in both protein partners and in this way facilitated analysis of the HDX‐MS results.

### Rab proteins adjust their conformation to REP upon complex formation

Rab proteins are globular molecules with an extended, unstructured C‐terminal part, the so‐called hypervariable region (Pylypenko et al., 2018). The globular part of Rabs contains the guanine nucleotide binding pocket: the amino acids forming the scaffold of the Rab molecule and those lining the interior of the GTP/GDP binding pocket are well conserved, while the Switch I and II regions and the P‐loop, all of which are engaged in coordination of guanine nucleotide phosphates and Mg^2+^ ions, are less conserved (Figure 4c). The hypervariable C‐terminal tail, containing the prenylatable cysteines at its far end, also shows weak conservation (Figure 4c).

In the H‐D exchange experiment, GST‐Rab‐F2b underwent robust changes upon complex formation with REP‐His, observed as a difference in H‐D exchange between the apo form and the complexed form (Figure 2a,b). No changes were seen in any of the GST tag peptides, covering the full length of the GST polypeptide chain (Figure S3), which confirms that only Rab‐derived peptides, engaged in a specific manner in the Rab–REP interaction, showed significant changes in the HDX‐MS experiment. This supports the notion that these changes reveal significant molecular events. In the REP‐His/GST‐Rab‐F2b complex after 10 sec of H‐D exchange, the large structural element built of amino acids 51–78 of Rab‐F2b became highly protected from the environment (Figures 2a,b and 4c). These amino acids build the Switch II/interswitch region in Rab‐F2b crystal structures (Figure S6b; Uejima et al., 2010). This structural change was accompanied by minor changes in H‐D exchange at the very N‐terminus of the protein (peptide 4–12), which also forms a part of Switch II (Figures 2a,b and 4c). Decreased solvent accessibility was also seen around amino acids 20–26, which form the P‐loop (Uejima et al., 2010) (Figures 2a,b and 4c). In the described experiment, we were unable to assign the correct MS spectra to a C‐terminal peptide of GST‐Rab‐F2b containing the prenylatable double‐cysteine motif. This might be due either to the low abundance of this highly hydrophobic peptide in the HPLC eluate prior to ESI‐MS or to its poor ionization ability. However, the most C‐terminal of all assigned peptides (183–189) showed very fast proton–deuterium exchange in both free and complexed form (Figure 2b) in accordance with the structural model (Figure S6b).

Unfortunately, not many GST‐Rab‐D1 peptide signals could be correctly assigned (Figure 2c, Figure S2c). Despite this, we were able to find peptides that were unequivocally protected in the REP‐His complex with GST‐Rab‐D1. Interestingly, some of these peptides corresponded to regions of increased H‐D exchange protection in the REP‐His complex with Rab‐F2b (for example peptide 62–70 localized in the Switch II/interswitch region; Figures 2c,d and 4c; Cai et al., 2008), while other peptides showed much better protection in the Rab‐D1 complex than in the Rab‐F2b complex (for example peptides from the 12–31 region, the P‐loop, and peptide 41–45 from the Switch I region; Figure 2c,d, compare with the alignment and the structural models in Figure 4c and Figure S6c).

For GST‐Rab‐E1d we were able to assign signals to a comparable number of peptides as for GST‐Rab‐D1 (Figure S2d). Again, peptides forming the Switch I, Switch II, and interswitch regions, spanning amino acids 52–76, showed high protection at all time points, similarly as peptides from the P‐loop, spanning amino acid positions 17–38 (Figure 2e,f; Itzen et al., 2006). The change of the conformation at the P‐loop (peptides spanning region 17–38) and in region 115–131 were particularly strong at the 48 h time point. The latter region is also involved in guanine nucleotide binding by Rabs. The most C‐terminal peptides that we could assign were derived from the region 189–198, which forms the flexible C‐terminal tail (see alignment and model, Figure 4c, Figure S6d). These peptides were exchanging protons fast and efficiently in both the apo and complexed forms.

### The structure of REP‐His changes upon binding to Rab proteins

According to the crystal structure of the mammalian protein, REP is built of two domains, the large domain I, which bind Rabs, and the smaller domain II, which binds prenyl groups and the RGT catalytic complex (Pylypenko et al., 2003; Rak et al., 2004).

In general, in our H‐D exchange experiment, the REP‐His surface was better protected in the Rab‐F2b complex than in free REP‐His (Figure 3a). Some peptides differed significantly in deuterium uptake (Figure 3b). The REP‐His amino acid region with the strongest H‐D exchange protection upon GST‐Rab‐F2b binding was spanning amino acids 303–323 at the 1 h time point (Figure 3a,b). Inspection of the alignment (Figure S4) and the model (Figure S6a) suggests that these amino acids form the RBP – the main site of interaction of REP with Rabs (Rak et al., 2004). Peptides spanning the region 140–151 underwent transient protection at 10 sec after D_2_O addition (Figure 3b). This region, although distant in amino acid sequence, also folds as part of the RBP (Figure S6a; Rak et al., 2004). A slightly weaker level of protection was detected also for amino acids 274–281 at the 10 sec time point (Figure 3b). This region is highly conserved in the alignments (Figure S4) and in the mammalian structure forms the entrance to the geranylgeranyl binding tunnel (Pylypenko et al., 2003). Peptides spanning the region 185–190 underwent transient protection at 10 sec after D_2_O addition (Figure 3b). According to the alignment (Figure S4) and previous reports on Arabidopsis REP (Hala et al., 2005) this region serves as the RGTA binding site. It must be stressed that all REP regions undergoing significant H‐D exchange protection had a high redundancy of peptide library coverage and the described changes were seen in overlapping peptides (Figure S2a).

For the C‐terminus of REP no significant change upon Rab‐F2b binding could be detected under the given conditions (Figure 3a,b). However, all peptides located close to the C‐terminus of REP (starting from amino acid 534 in the sequence) underwent very fast H‐D exchange, suggesting that they were not engaged in any secondary or tertiary structures of the protein, neither in the apo nor in the complexed form (Figure 3b). This effect correlated well with predictions that the C‐terminus of REP does not form any secondary structure elements (Figure S5, Figure S6a).

We have also performed the HDX‐MS experiment for the REP‐His/GST‐Rab‐D1 and REP‐His/GST‐Rab‐E1d complexes. These two proteins showed weaker binding to REP‐His in the dot‐blot assay (Figure 1a,b) and also the obtained HDX‐MS results were less clear than for the Rab‐F2b complex, but still they showed that all three REP–Rab complexes induced structural changes in the same regions of REP (Figure 3), though in the REP–Rab‐D1 complex these changes were much weaker. In the amino acid region 274–281 (putative geranylgeranyl binding site) strong protection was detected at early time points for the REP‐His/GST‐Rab‐E1d complex (Figure 3f) and slighter protection for the REP‐His/GST‐Rab‐D1 complex (Figure 3d). In the region 185–189 (RGTA binding site) a decrease in proton exchange was visible at the early time points for REP‐His bound to GST‐Rab‐D1, followed by an increase at later time points, similarly as in the REP‐His/GST‐Rab‐F2b complex (Figure 3d), but the effect was not that pronounced in the case of REP‐His/GST‐Rab‐E1d (Figure 3f).

For the C‐terminus of REP, again fast H‐D exchange was detected, both for REP‐His/GST‐Rab‐D1 and for REP‐His/GST‐Rab‐E1d (Figure 3c–f). Interestingly, peptides localized in the 303–323 and 140–148 regions (RBP) were protected to a much lower extent in the REP‐His/GST‐Rab‐D1 complex than in the REP‐His/GST‐Rab‐F2b complex (Figure 3b,d). On the contrary, the 302–323 region at later time points became more protected in the REP‐His/GST‐Rab‐E1d complex than in REP‐His/GST‐Rab‐F2b, but no change was seen for the amino acids spanning the 140–148 region (Figure 3b,f). Additionally, the N‐terminally localized peptide spanning the region 1–14 and peptides in the region 478–489 in REP‐His were protected upon binding of GST‐Rab‐E1d (Figure 3f).

The changes detected in all investigated REP–Rab complexes were thus localized mainly in the same regions of REP, but binding of Rab‐F2b and Rab‐E1d induced more pronounced changes than binding of Rab‐D1, in particular in the RBP surface. The C‐terminus of REP seemed to be uncomplexed in all analyzed plant REP–Rab structures, contrary to the situation in the mammalian complex, where the REP‐1/Rab7 C‐termini align in an anti‐parallel arrangement in the crystal structure (Rak et al., 2004).

The presented data for plant REP–Rab complexes are thus contradictory to the model of mammalian REP–Rab complex formation, where the C‐termini of both proteins were shown to be indispensable for Rab geranylgeranylation, with a single‐residue mutation in a hydrophobic amino acid in the Rab7 C‐terminus (Ile 192 from the PIKL motif) precluding its lipidation (Guo et al., 2008; Wu et al., 2009). It was elegantly shown by introducing single and multiple mutations in the Rab C‐terminal hypervariable tail followed by a detailed biochemical analysis that mammalian REP and Rab C‐termini interact (Guo et al., 2008). Computational models supported the notion (Wu et al., 2009), but the consequences of the lack of an interaction between the REP and Rab C‐termini were never addressed *in vivo* in the mammalian system. In our HDX‐MS experiment, the peptides containing equivalent residues (Met 183 in the MVLP motif of Arabidopsis Rab‐F2b and Ile 191 in the GIKI motif of Rab‐E1d, see alignment in Figure 4c) were highly unprotected both in the apo form and in complex with REP, and presumably they were not engaged in any protein–protein interaction. To better understand the role of the REP C‐terminal tail in plant cells, we turned to genetic analysis.

### A truncated REP variant lacking the C‐terminal tail is sufficient for plant viability

The presented biophysical results encouraged us to study the consequences of mutations in the C‐terminal part of the *A. thaliana* REP protein. We analyzed two *A. thaliana* lines carrying transfer DNA (T‐DNA) insertions in the 3′ end of the *REP* gene: SALK_140044, named *rep*‐*1*, was the only viable line with insertion in the *REP* gene, while GABI‐Kat_295F01, named *rep*‐*2*, was unable to produce viable homozygotes. The *rep‐1/rep‐1* and *REP/rep‐2* plants were similar to WT plants grown in parallel (Figure S7a).

We reasoned that the lack of homozygous plants in the progeny of the *REP/rep‐2* parent could be due to the sterility of gametes or to embryolethality. We tested 260 progeny of *REP/rep‐2* plants, and half of them were WT and the other half were *REP/rep‐2*. Statistical analysis of these results supported the hypothesis that one of the gametes was non‐functional (Table 1). No obvious disturbances in ovule development were detected, and a reciprocal backcross of *REP/rep‐2* to WT revealed a male transmission block (Table 1). This suggests that the male transmission defect in *rep*‐*2* is of gametophytic origin. Analysis of 89 progeny obtained through self‐pollination of *REP/rep‐1* plants suggested a milder transmission defect in this line as well (Table 1). Again, a backcross to WT plants showed that transmission through the male lineage was impaired (Table 1). We also performed a genetic cross between both mutants (*REP/rep‐2* was pollinated with *rep*‐*1/rep‐1)*. From this cross we obtained viable, fertile plants, carrying in the *REP* locus one copy of the *rep‐1* allele and one copy of the *rep‐2* allele (Figure S7a,b). This showed that one copy of the *rep‐1* allele was sufficient for plant survival.

**Table 1:** Genetic analysis of mutated *REP* allele transmission

cross	Expected ratio WT:het:hom	Observed WT:het:hom	Number of observations	*P*‐value	Significance
*REP/rep‐1* (self)	1:2:1	29:46:14	89	0.2529	NS
WT ♀ × *REP/rep‐1 ♂*	1:1:0	76:20:0	96	<0.0001	^***^
*REP/rep*‐*2* (self)	1:2:1	124:136:0	260	<0.0001	^***^
*REP/rep*‐*2* ♀ × WT ♂	1:1:0	46:53:0	99	0.7761	NS
WT ♀ × *REP/rep‐2 ♂*	1:1:0	49:0:0	49	<0.0001	^***^

*REP/rep‐2* and *REP*/*rep‐1* plants were either left for self‐pollination or manually crossed to WT as pollen donors or acceptors, as stated in the table. Progeny coming from the crosses was grown in soil for 4 weeks, genomic DNA was isolated from leaves, and plants were genotyped by PCR with appropriate primer pairs (Table S2). Results were analyzed by the χ^2^ test or the Fisher exact test against the H_0_ hypothesis that the genetic segregation is Mendelian. ***P < 0.001.

To ensure that the pollen defects of *REP/rep‐2* and *REP/rep‐1* plants are attributable only to the mutations in the *REP* locus, we transformed the *35S:REP‐GFP* construct into *rep‐1/rep‐2* mutant plants by the floral dip method. In the next generation of plants we obtained viable *rep‐2/rep‐2* plants expressing the construct, indistinguishable from the WT plants (Figure S7c,d). The segregation of the *rep‐1* and *rep‐2* alleles as well as of the *REP‐GFP* construct in the progeny of an F1‐generation *rep‐1/rep‐2 35S:REP‐GFP* plant is shown in Table S1. The results are consistent with the assumptions that the full sterility of *rep‐2* pollen and the partial deficiency of *rep‐1* pollen are both reversed by the expression of REP‐GFP. In parallel we transformed WT plants with the construct and proved that the recombinant protein localizes to the cytoplasm and intracellular vesicular structures (Figure S7e).

### The C‐terminally truncated REP protein is present in the *rep‐1* mutant

**Figure 5:**
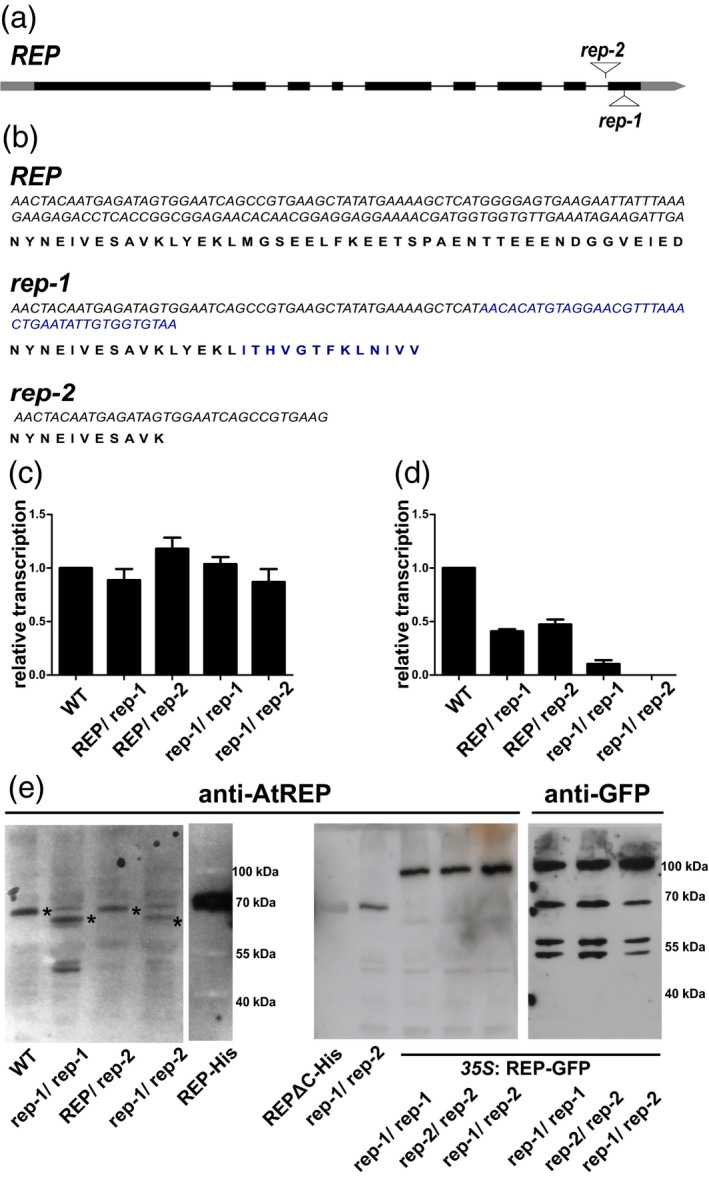
Characteristics of *rep* mutants in *Arabidopsis thaliana*. (a) Gene model of the *REP* open reading frame, where boxes represent exons, connecting lines represent introns, and sites of T‐DNA insertions are marked. (b) Nucleotide and (predicted) amino acid sequences of the last two exons of REP in the WT and *rep*‐*1* mutant lines. Blue letters denote the modified 3′ gene region and the corresponding C‐terminal fragment of the *rep‐1* allele‐encoded protein, due to the insertion of T‐DNA. Precocious termination of translation in *rep‐1* is caused by two consecutive stop codons. The *rep‐2* allele carries the T‐DNA insertion in the last intron; shown is only the nucleotide sequence of the previous exon and its translation. (c, d) RT‐qPCR analysis of the *REP* gene in WT, *REP/rep‐1*, *REP/rep‐2*, *rep‐1/rep‐1*, and *rep‐1/rep‐2* plant lines. Similar analysis for a *rep‐2/rep‐2* line could not be performed due to the lack of homozygous plants. (c) RT‐qPCR analysis with a primer pair amplifying a region of the *REP* gene upstream of both T‐DNA insertions (primers REP‐F3 and REP‐R4; see Table S2). (d) RT‐qPCR analysis with a primer pair amplifying the C‐terminal region of the *REP* gene, which is absent or modified in the *rep‐1* and *rep‐2* alleles (primers REP‐F5 and REP‐R6; see Table S2). (e) Western blot analysis with mouse polyclonal anti‐AtREP as primary antibody. Cytosolic protein fractions from WT, *rep*‐*1/rep‐1*, *REP/rep*‐*2*, and *rep*‐*1/rep*‐*2* lines as well as from revertant plants, *rep‐1/rep‐1*, *rep‐2/rep‐2*, and *rep‐1/rep‐2* overexpressing REP‐GFP under the control of the CaMV *35S* promoter, were prepared from rosette leaves of 4‐week‐old plants. Purified recombinant REP‐His and REPΔC‐His were used as controls. Plants carrying the *CaMV35S:REP‐GFP* construct were also probed with an anti‐GFP antibody. Equal amounts of total protein were loaded in each lane, apart from the recombinant proteins used as controls. Asterisks on the left panel mark REP protein variants. Note the non‐specific band migrating at a similar molecular weight as WT REP. Images of all mutant and revertant plants are shown in Figure S7.

In the *rep*‐*1* mutant the T‐DNA insert was localized in the last, ninth exon of the gene (Figure 5a) and it was predicted to cause a frameshift resulting in the replacement of 30 native C‐terminal amino acids with 13 novel ones (Figure 5b). This amino acid stretch spans the entire C‐terminal tail of REP, which is predicted to be unstructured and whose removal, judging from the model, is likely not to preclude formation of any of the well‐defined structural motifs (compare Figures S5 and S6a). The predicted sequence of the *rep*‐*1* C‐terminus does not contain any negatively charged residues, while the WT version contains 12 acidic residues out of 30 (Figure 5b). In the *rep‐2* line the T‐DNA insertion was placed slightly upstream of the *rep‐1* mutation, in the last intron of the *REP* gene (Figure 5a). This truncation removes 35 C‐terminal amino acids, comprising the tail fragment and part of the predicted last helix (Figures S5 and S6a).

To investigate why one truncation left the plants viable (*rep‐1*) and the other was detrimental *in vivo* (*rep‐2*), we used quantitative real time‐PCR (RT‐qPCR) analysis to look at the expression of both alleles. As expected, the full‐length *REP* transcript was present in half the WT amount in *REP/rep‐1* and *REP/rep‐2* plants and was nearly absent from *rep‐1/rep‐1* and *rep‐1/rep‐2* plants (Figure 5d), but PCR with primers designed to amplify a fragment upstream of the T‐DNA insertions confirmed that the large N‐terminal part of the gene was transcribed in all tested lines (Figure 5c). The *rep*‐*2* allele was thus transcribed at a similar level as the *rep*‐*1* allele.

To establish whether the truncated *REP* transcripts undergo translation in the *rep‐1* and *rep‐2* mutants, we performed Western blot analysis of protein extracts from the leaves of WT, *rep‐1/rep‐1*, *REP/rep‐2*, and *rep‐1/rep‐2* plants as well as from *rep‐2/rep‐2* and *rep‐1/rep‐2* plants carrying the *35S:REP‐GFP* construct (Figure 5e). For protein detection we used an anti‐AtREP antibody, a gift from Dr. Michal Hala, Charles University, Prague. This antibody recognized both the WT version of REP present in the WT and *REP/rep‐2* lines and the truncated version present in the *rep‐1/rep‐1* and *rep‐1/rep‐2* lines (Figure 5e, left panel). The truncated version is 17 amino acids shorter, but it showed visibly higher electrophoretic mobility, likely due to the lack of 12 negatively charged residues. No additional truncated protein band was detected in the *REP/rep‐2* line, suggesting that the *rep‐2* allele might not lead to the production of a stable protein. The antibody also detected the REP‐GFP protein (middle panel). Surprisingly, in the lines expressing REP‐GFP the fusion protein was the only version of REP recognized, and the truncated protein encoded by *rep‐1* was not visible. The presence of the fusion was also confirmed by probing the blot with an anti‐GFP antibody (right panel).

Taken together, these data show that in the *rep‐1* line there is a truncated version of the REP protein present and that it is sufficient to support plant viability. For these reasons, we decided that the *rep‐1* line was a suitable model to study the influence of the REP C‐terminus on Rab binding and geranylgeranylation *in planta*. The absence of an additional higher‐mobility band in the *REP/rep‐2* line suggests that the *rep‐2* allele does not code for a stable version of the REP protein. Combined with the fact that *rep‐2* homozygotes were non‐viable, these data led us to consider the *rep*‐*2* allele as equivalent to a functional knock‐out, i.e., completely lacking the REP function.

### Pollen development and pollen tube growth are affected in lines carrying the *rep‐1* and *rep‐2* alleles

Initial phenotypic investigation of the *rep‐1/rep‐1* and *REP/rep‐2* insertional lines showed no striking differences when compared to WT plants, neither in vegetative nor in generative organs (Figure S7a).

**Figure 6:**
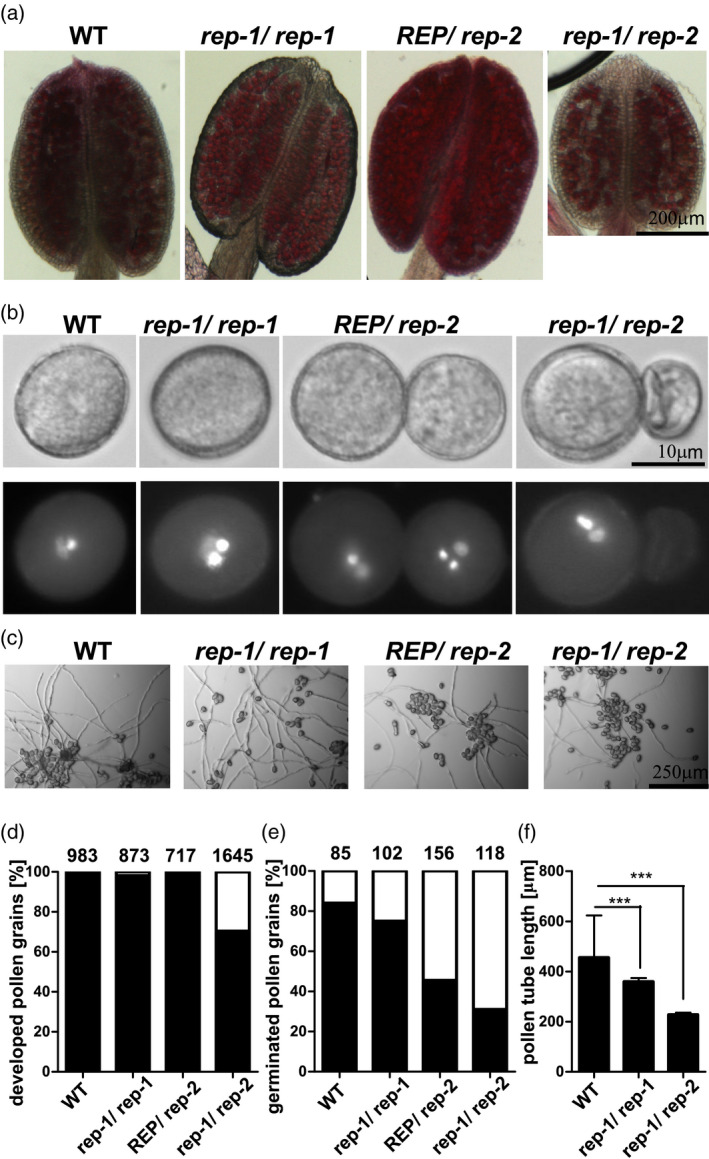
Pollen development and germination in *rep* mutants. (a) Mature anthers of WT and *rep* mutant plants were stained with Alexander stain for analysis of pollen viability. Purple‐stained grains are viable. In *rep‐1/rep‐2*, empty spaces and black remnants of pollen grains are visible in the anther. Bar = 200 μm in all pictures. (b) Mature pollen grains of WT and *rep* mutant plants were stained with DAPI to visualize DNA. Upper panel, DIC microscopy; lower panel, fluorescence microscopy. Bar = 10 μm in all pictures. (c) *In vitro* pollen germination of pollen derived from WT and *rep* mutant plants. Pollen was germinated on solid medium for 16 h in the dark. Bar = 250 μm in all pictures. (d) Fraction of normally developed pollen grains from WT and *rep* mutant plants [%]. Pollen from mature anthers was spilled on solid medium. Black bars, pollen grains of normal shape and size; white bars, shrunken pollen grains. Numbers above bars show the number of pollen grains counted. (e) Fraction of WT and *rep* mutant pollen grains forming pollen tubes [%]. Pollen from mature anthers was spilled on solid medium and left for germination for 16 h in the dark. Black bars, pollen grains forming tubes of at least 5× pollen diameter; white bars, pollen grains not forming pollen tubes. Numbers above bars show the number of pollen grains counted. f) Length of pollen tubes formed by pollen grains derived from WT and *rep* mutant plants. Tube length was measured after 16 h of *in vitro* growth; bars represent mean length ± SD, ***P < 0.001 in a one‐sided Student *t*‐test. More than 50 pollen tubes were counted for each genotype; the experiment was repeated three times with similar results. Corresponding data for pollen coming from revertant plants is shown in Figure S8.

To gain further insight, we investigated the generative organs of *rep‐1/rep‐1*, *REP/rep‐2*, and *rep‐1/rep‐2* mutants. We stained mature anthers of the assayed plants with Alexander stain. The anthers of WT, *rep‐1/rep‐1*, and *REP/rep‐2* plants were full of viable pollen grains (Figure 6a). The *rep‐1/rep‐1* and *REP/rep‐2* lines displayed no changes in pollen grain shape or viability, despite the sterility of *rep*‐*2* pollen in genetic crosses. In contrast, the anthers of *rep‐1/rep‐2* plants were smaller and empty spaces between grains were visible. We then stained nuclei in the grains by means of DAPI. Mature pollen grains of WT, *rep‐1/rep‐1*, and *REP/rep‐2* plants were almost exclusively trinuclear (Figure 6b). For *rep‐1/rep‐2* plants, a significant fraction (30%) of pollen grains were shrunken and did not contain DNA (Figure 6a,b,d). The degradation of DNA and pollen degeneration at early developmental stages suggests that intact REP activity in maternal sporophytic tissues of the anthers is necessary for correct pollen development. The (modest) *rep‐1* male transmission defect may thus be of sporophytic origin.

We also assayed *in vitro* pollen germination. Here we found a considerable decrease in pollen germination in the *REP/rep‐2* and *rep‐1/rep‐2* lines, and only a slight effect in the *rep‐1/rep‐1* line (Figure 6c,e). These results fit well with the genetic data, which shows a lack of transmission of the *rep‐2* allele through the male germline and a partial defect in male transmission for the *rep‐1* allele (Table 1). When we measured the length of the germinated pollen tubes, we found a decrease in tube length for pollen derived from *rep‐1/rep‐1* plants and, even more pronounced, for pollen derived from *rep‐1/rep‐2* plants (Figure 6f), again suggesting sporophytic influence on pollen fitness by truncated REP present in the *rep*‐*1* mutant. Expression of the *35S:REP‐GFP* construct in the *rep‐1/rep‐2* line reversed the pollen deformation phenotype (Figure S8a,b).

### Ultrastructural analysis of *rep‐1* mutant tissues reveals minor changes in cell structure

The *S. cerevisiae mrs6* mutant accumulates an increased number of unfused vesicles as well as highly proliferated endoplasmic reticulum (ER) membranes (Jiang and Ferro‐Novick, 1994), similar to the *bet2* mutant in *RGTB* (Newman and Ferro‐Novick, 1987; Rossi et al., 1991). To see if the same kind of ultrastructural changes, related to reduced Rab function, could be found in the Arabidopsis *rep‐1* mutant, we performed transmission electron microscopy (TEM) analysis of root, stem, and leaf tissues.

**Figure 7:**
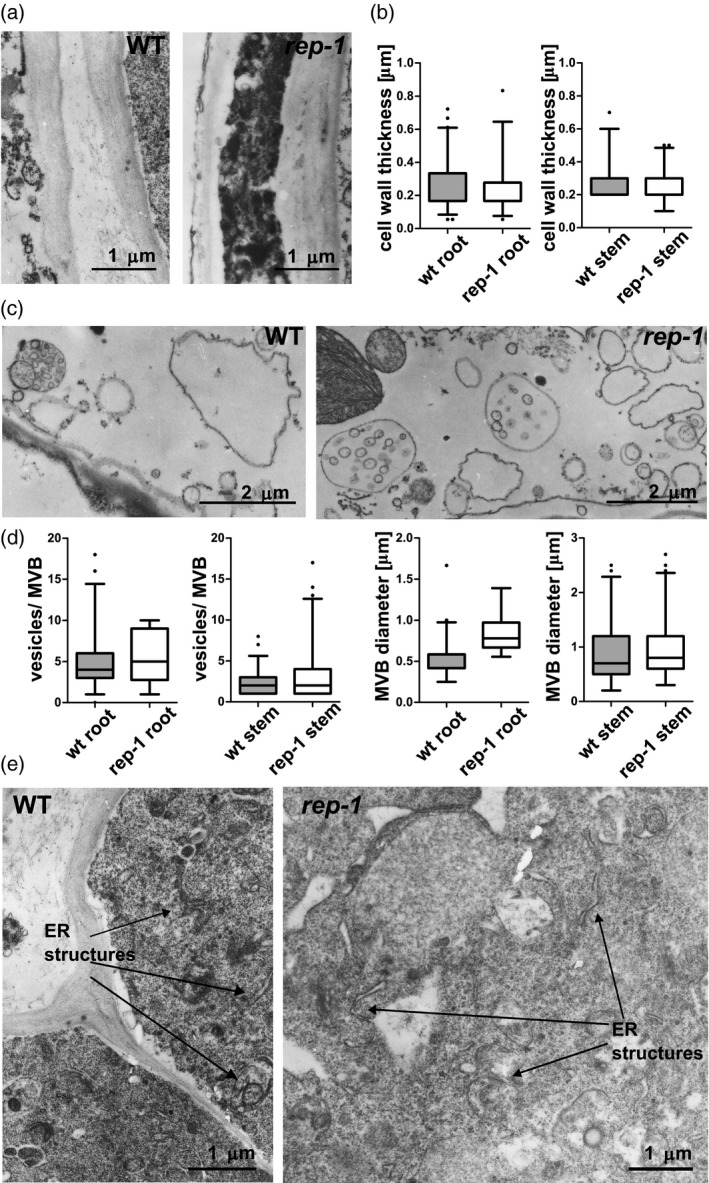
TEM ultrastructural analysis of sporophytic tissues of the *rep‐1* mutant. (a) No changes could be detected in the cell wall of root parenchymal cells. More than 20 images of four to five cells each were analyzed for each genotype. Scale bar = 1 µm for both images. (b) Quantification of cell wall thickness in *rep*‐*1* parenchymal root and stem cells. The mean value is marked; boxes represent 25% and 75% percentiles and whiskers represent the 95% confidence interval. (c) Multivesicular bodies (MVBs) in stem (c) and root (d) parenchymal cells. More than 10 images of four to five cells each were analyzed for each genotype. Scale bar = 2 µm for images of stem cells (c) and scale bar = 1 µm for images of root cells (d). (e) Quantification of MVB diameter and number of vesicles per MVB in root parenchymal cells and stem parenchymal cells of the *rep‐1* mutant. MVBs in parenchymal root cells seemed more abundant and larger and contained more vesicles in their lumen in the *rep‐1* mutant than in the WT. No differences in MVBs could be detected between WT and *rep‐1* cells from stems. The mean value is marked, boxes represent 25% and 75% percentiles, and whiskers represent the 95% confidence interval. (f) ER structures in root parenchymal cells do not differ between the WT and *rep‐1* lines. A representative image of 5–10 electronographs is presented for each genotype. Scale bar = 1 µm in (e). Complementary results for other organelles are shown in Figure S9.

First we analyzed structures potentially connected to the functioning of the Rab proteins investigated in this work. For the cell membrane and the cell wall, whose correct biogenesis depends on the Rab‐E family (Speth et al., 2009), no significant changes were detected in cell wall thickness in both root and shoot parenchyma (Figure 7a,b). Also ER and Golgi morphology, which are dependent on the Rab‐D family (Pinheiro et al., 2009), seemed unchanged (Figure 7f, Figure S9a). Only multivesicular body (MVB) morphology, dependent on the Rab‐F family (Ito et al., 2016), displayed minor changes. In root parenchymal cells, the number of vesicles in the lumen of the MVBs was slightly higher in mutant than in WT cells and the diameter of the compartments was clearly increased; in stem parenchyma cells no changes were detected (Figure 7c–e). These effects are in accordance with the notion that *rep‐1* cells display minor changes in Rab functioning.

In root cells we also observed that occasionally *rep‐1* cells contained clusters of oil bodies (Figure S9b) or an increased number of transport vesicles (Figure S9d), resembling the yeast *mrs6* phenotype mentioned above. Although vacuolar morphology was unchanged in leaves (Figure S9c), we unexpectedly found increased accumulation of starch granules and plastoglobules in the chloroplasts of *rep‐1* mutant leaf cells (Figure S9e,f).

### Prenylation of Rabs is only weakly affected in the *rep‐1* mutant

The lack of a clear phenotype under typical growth conditions in the *rep‐1* line suggested that overall Rab geranylgeranylation was not reduced substantially. However, changes in the ultrastructure of selected membrane compartments (MVBs, transport vesicles) in the *rep*‐*1* mutant would be consistent with some Rab proteins being affected. This hypothesis would be in line with the biochemical results which showed that some Rabs interact with REP more strongly than others (Figure 1a).

**Figure 8:**
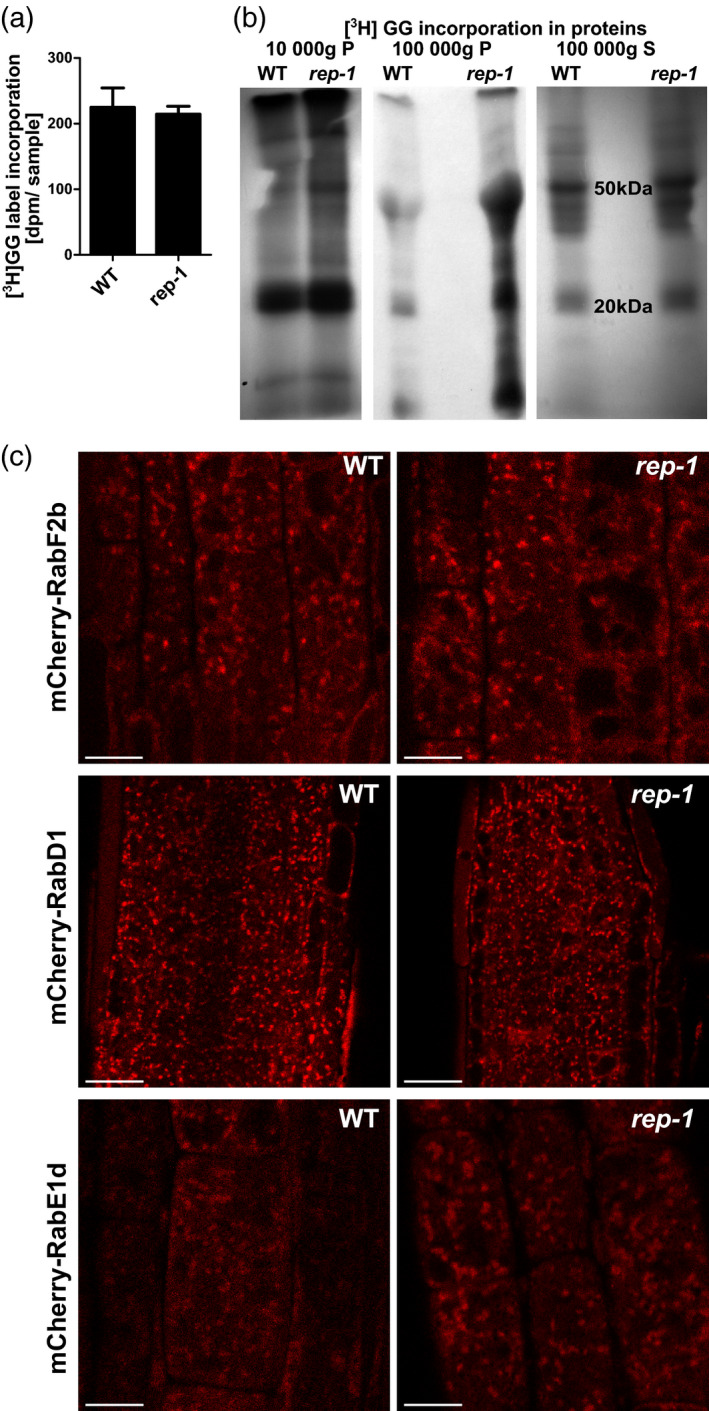
Rab protein prenylation and localization in the *rep*‐*1* mutant. (a) Quantification of *in vivo* metabolic incorporation of [^3^H]geranylgeraniol in plant proteins with a molecular mass of 17–30 kDa. Total lysates were prepared without fractionation and resolved by SDS‐PAGE. Gel regions corresponding to the size of Rab proteins (17–30 kDa) were cut out and solubilized, and their radioactivity was quantified in a scintillation counter. Bars represent the mean of at least three independent biological experiments ± SD. (b) *In vivo* metabolic incorporation of [^3^H]geranylgeraniol in plant proteins. Total lysates were prepared from seedlings of WT and *rep‐1/rep‐1* lines cultured on medium containing [^3^H]geranylgeraniol. Lysates were separated into three fractions, 10 000 ***g*** pellet, 100 000 ***g*** pellet, and 100 000 ***g*** supernatant, resolved by SDS‐PAGE, and analyzed by autoradiography. The results of a representative experiment are shown. Fractions from the 100 000 ***g*** pellet showed much higher [^3^H]GG incorporation, and lower exposure of the same gel is shown. In all lanes except the *rep‐1* 100 000 ***g*** pellet a similar amount of total protein per lane was loaded; in the *rep‐1* 100 000 ***g*** pellet lane a higher amount of protein was loaded. (c) Localization of selected Rab proteins in the *rep*‐*1* background. mCherry fusions of Rab‐F2b (upper panel), Rab‐D1 (middle panel), and Rab‐E1d (lower panel) were introduced into the *rep‐1*/*rep‐1* line by crossing. Localization in root epidermal cells (upper and lower panels) and root meristematic cells (middle panel) is shown. CSLM images, scale bar = 10 µm for upper and lower panels and scale bar = 20 µm for the middle panel.

To check if indeed some Rabs were hypoprenylated in the *rep*‐*1* mutant, we applied an *in vivo* labeling method (Gutkowska et al., 2004). [^3^H]‐labeled geranylgeraniol was added to plant growth medium. The compound was shown to be effectively taken up and used for protein lipidation. When compared to WT plants, the *rep‐1* mutant displayed only a negligible decrease in incorporation of the [^3^H]GG‐OH precursor, suggesting that the C‐terminal truncation of REP did not decrease overall RGT activity (Figure 8a,b).

In order to see if also Rab localization is maintained in the *rep‐1* mutant, we chose the three Rabs that we used in the H‐D exchange experiment and introduced their fluorescently tagged versions into the *rep‐1* background. These Rabs come from different subfamilies and show different subcellular localization (Geldner et al., 2009). Rab‐E1d is an exocytosis‐related protein, labeling vesicles present in the vicinity of the plasma membrane (Geldner et al., 2009), but is found also in the Golgi apparatus and in the plasma membrane (Camacho et al., 2009; Speth et al., 2009; Zheng et al., 2005). Rab‐F2b is an endocytosis‐related protein present on late endosomes in various cell types (Ito et al., 2016; Kotzer et al., 2004; Lee et al., 2004). Rab‐D1 is a protein mediating transport out of the ER to the Golgi apparatus (Pinheiro et al., 2009; Zheng et al., 2005), but is also found on endosomes (Geldner et al., 2009). As observed in meristematic and epidermal cells of seedling roots, all of these Rab GTPases retained clear association with endomembrane compartments in the *rep‐1* background, similarly as in WT cells (Figure 8c).

## DISCUSSION

### Rab binding induces structural changes in REP

In this work we set out to investigate the plant REP–Rab interaction in a more detailed manner than has been attempted previously. We wanted to know which parts of the proteins are involved in the interaction and if this is the same for Rab proteins coming from different subfamilies. In particular, we were interested if the C‐terminal tail of plant REP is directly engaged in the interaction with Rab GTPases. We also wanted to know the dynamics of the interaction. How much structural change occurs in REP upon complex formation, and in which regions? Does this depend on the type of Rab bound? And conversely, what changes are induced by REP in the Rab regions that are unavailable for crystallographic studies due to high flexibility, for example the C‐terminal hypervariable tail or the GDP/GTP binding site (Guo et al., 2008; Pereira‐Leal et al., 2003; Rak et al., 2003, 2004; Seabra, 1996; Wu et al., 2009)?

REP sequences are similar among representatives of one kingdom of life, but differ largely between distant groups of organisms (Rasteiro and Pereira‐Leal, 2007). Large regions containing amino acid residues important for binding of Rab proteins (RBP) or of RGTA/RGTB heterodimers are well conserved between animal, yeast, and plant REPs. In particular, the RBP shows high conservation, but interestingly, the H‐D exchange protection in this region is higher in the REP–Rab‐F2b and REP–Rab‐E1d complexes than in the REP–Rab‐D1 complex. This may reflect the different affinity of these Rabs for REP and it is possibly mirrored by the engagement of different structural motifs of the two Rabs during complex formation with REP. Indeed, in Rab‐F2b mainly the Switch II/interswitch region showed protection upon REP binding, while in Rab‐D1 the most prominent changes were observed in the P‐loop, which coordinates GDP phosphates. In Rab‐E1d both regions were protected upon complex formation with REP. Corresponding results were obtained during studies on different yeast Rabs interacting with the GDI molecule, where each Rab preserved the general fold but the flexible switch regions gave different contributions to GDI binding (Ignatev et al., 2008). It suggests that individual regions in Rabs differ in their relative contribution to protein partner binding, a situation similar to Rab binding to GEFs, which can achieve highly selective recognition of distinct subsets of Rab GTPases exclusively through interactions with the Switch and interswitch regions (Eathiraj et al., 2005). Quite similarly, the GDI protein from yeast and mammals, related structurally and functionally to REP, binds Rab molecules with different affinities (*K*d value range spanning two orders of magnitude), although engaging the same binding surface for interaction with different Rabs (Pylypenko et al., 2006; Rak et al., 2004). Yeast REP was used in the same study for comparison and it was shown again that the Rab‐binding interface on the REP molecules is more conserved than the REP‐binding interface on Rabs (Pylypenko et al., 2006). These results support the hypothesis that REP binds a relatively diverse subset of Rab proteins recognizing them not by their prenylation consensus sequence as other protein prenyltransferases do, but by Rab subfamily‐specific surface epitopes.

We also wanted to answer the question whether the C‐terminal fragment of plant REP, corresponding to the amino acid residues deleted in REPΔC‐His and truncated in *rep‐1*, takes part in the REP–Rab interaction. In animal REP, this part of the protein is not responsible for strong binding to Rabs, but it facilitates the positioning of the Rab C‐terminus (containing the prenylatable cysteines) towards the RGT active site (Guo et al., 2008; Rak et al., 2004). In the absence of a bound Rab this fragment is engaged in REP homodimer domain swapping, at least in the crystal structures (Pylypenko et al., 2003; Rak et al., 2004). Plant REP sequences are similar to each other in this region, but their similarity to yeast and animal REPs is very weak here.

Using HDX‐MS we examined if this particular fragment is protected in the REP–Rab complex when compared to the apo state. All peptides obtained from this region exchanged amide protons with the environment at a high rate. This suggests that the region is highly flexible and has few secondary structure elements, which is in accordance with our theoretical predictions. In our experimental conditions we were unable to see any binding of the REP C‐terminal tail to unprenylated Rab‐F2b, Rab‐D1, or Rab‐E1d, not even transient. Consistent with this, three independent peptides derived from the putative REP C‐terminus‐binding region of Rab‐F2b and two corresponding peptides from Rab‐E1d did not show any changes in H‐D exchange upon complex formation.

These results have surprised us in light of the biochemical and crystallographic data for the mammalian REP–Rab7 complex (Guo et al., 2008; Rak et al., 2003; Wu et al., 2009), which showed the C‐terminus of REP to be indispensable for Rab geranylgeranylation. However, these results are not necessarily contradictory. The C‐terminal part of plant REP may be involved in the binding of Rabs from other subfamilies than we have used in our experiments. Which Rab proteins or subfamilies would be dependent on WT REP activity in plants is yet to be shown. Another possibility would be that the C‐terminus of plant REP may be engaged in the binding of (mono)geranylgeranylated Rab proteins. It is known that monoprenylated Rabs bind to REP with about 20 times higher affinity than their unprenylated forms (63 pm for mono‐geranylgeranylated versus 1 nm for unprenylated mammalian Rab7; Shen and Seabra, 1996; Wu et al., 2007). Finally, it is conceivable that the REP C‐terminal tail binds a yet unidentified interactor. If this was the case, then the C‐terminus of REP could be involved in other functions than Rab prenylation. One possibility would be that it influences REP association with membranes and consequently its subcellular localization. In yeast, C‐terminal truncation of REP enhanced its affinity for membranes (Miaczynska et al., 1997), and the lack of negative charge on the C‐terminal tail of the plant REP, as in the *rep‐1* mutant, may facilitate binding of the negatively charged membrane lipids, but this hypothesis awaits further experimental proof.

### *In vivo* effects of REP C‐terminal truncation in plants

A knock‐out of REP activity is lethal in all eukaryotes studied so far (Bauer et al., 1996; Jiang and Ferro‐Novick, 1994; Moosajee et al., 2009; Shi et al., 2004; Thole et al., 2014). In plants a knock‐out of the *REP* gene has been described in a haploid moss, *P. patens* (Thole et al., 2014). Similar to the single knock‐outs in the Rab prenylation machinery gene *RGTA* and double knock‐outs in *RGTB* genes, the *rep* mutant of *P. patens* was non‐viable.

In the light of these data a *REP* knock‐out in Arabidopsis was likely to be non‐viable as well. Here we present two closely located mutations in the *REP* gene of Arabidopsis showing strikingly contradictory phenotypic manifestations. The *rep*‐*2* mutation is pollen sterile, but the *rep*‐*1* mutant is not affected profoundly in the sporophytic generation, a situation resembling the *Caenorhabditis elegans* and human *CHM* cases (Andres et al., 1993; Tanaka et al., 2008). In case of both mutants the transcription of the full‐length *REP* gene is lost, but both truncated transcripts are present in amounts comparable to WT. However, the *rep*‐*1* allele is translated into a protein of nearly preserved functionality, while the *rep*‐*2*‐encoded protein is not detectable in plant extracts. We explain these opposing results by the secondary structure of the REP protein at the site of the deletions. Apparently, the *rep*‐*1* mutation deprives the protein of the highly mobile, unstructured tail (Figure S10, a model built on PHYRE^2^). A similar truncation was recently described in the maize (*Zea mays*) GDI protein and it also left the protein active (Liu et al., 2020). On the contrary, the *rep*‐*2* mutation destroys the structure of the α‐helix aligned along and probably stabilizing the vast β‐sheet of the larger REP subdomain (Figure S10). This probably disturbs the tertiary protein structure and causes aggregation or degradation of the non‐functional protein, leading to gamete sterility.

The REP protein, together with the catalytic heterodimer RGTA/RGTB, is part of an enzymatic complex exerting Rab geranylgeranyl transferase activity. Mutations in *RGTB* genes in Arabidopsis have been shown to cause severe abnormalities in the development of both the sporophyte and the gametophytes (Gutkowska et al., 2015; Hala et al., 2010; Rojek et al., 2021a,b). An *rgtb1 rgtb2* double mutant, devoid of RGTB activity, is lethal due to male gametophyte sterility (Gutkowska et al., 2015) and this effect is of sporophytic origin. It seems likely that a similar situation can be observed with *rep‐1/rep‐2*‐derived pollen. For *rgtb1* pollen, when it is derived from *RGTB1/rgtb1* plants, it is fertile, but the pollen tubes are frequently branched or swollen (Gutkowska et al., 2015). In the case of *rep‐1/rep‐1* pollen we did not notice such defects, but the length of pollen tubes and the transmission efficiency through the male germline were decreased in comparison to WT pollen. *REP/rep‐2*‐derived pollen displayed defects of medium strength when compared to pollen from *rep*‐*1/rep‐1* and *rep‐1/rep*‐*2* plants; it was unfertile, but the pollen grains were viable and not deformed.

The reason why the female gametophyte is not affected in *rep‐1* or *rep‐2* lines may be that vesicular traffic is not so active and the flux of nutrients and hormones relies mainly on the sporophytic tissues of the ovule (Rojek et al., 2021a). Similarly, the development of pollen grains in the anthers, which is also dependent on the sporophytic tissues, is not strongly affected in *rep‐1* and *rep‐2* lines. On the contrary, pollen tube germination and growth, processes engaging vigorous vesicular transport and membrane recycling and dependent solely on the gametophyte itself, are highly limited in *rep‐1* and *rep‐2*. Introduction of the REP‐GFP fusion into the *rep‐1/rep‐2* plant reversed the male transmission defect.

In the *rep‐1* mutant the number and clustering of some membrane‐surrounded compartments was increased in comparison to WT plants. This was true for vesicles inside MVBs, transport vesicles, and oil bodies. However, other compartments, in particular the ER and vacuoles, did not show any changes. This supports our hypothesis that in the *rep‐1* line only some – maybe not very numerous – Rabs are affected by the REP C‐terminus deletion. The nature of the ultrastructural changes (vesicles gathering in cytoplasm and in MVBs) is consistent with mild defects in Rab‐regulated membrane fusion events.

Interestingly, also intrachloroplastic changes were obvious in *rep‐1*, such as accumulation of starch granules and lipid storage plastoglobules. This finding is surprising, since chloroplasts are typically thought to be excluded from Rab‐mediated traffic. On the other hand, inhibiting the post‐Golgi/early endosome recycling by Brefeldin A causes starch accumulation in Arabidopsis and *Chlamydomonas* chloroplasts (Hummel et al., 2010). The same drug also causes accumulation of triacylglycerols in plastoglobules of *Chlamydomonas*, similarly to what we observed in the *rep‐1* Arabidopsis mutant (Kato et al., 2013).

### C‐terminal truncation of REP in plants affects only some Rab proteins and Rab‐dependent processes

Since the C‐terminal REP tail that is lacking in the *rep‐1* protein is not universally conserved, we expected that basic REP activity could be preserved in the mutant lines, possibly with altered Rab specificity or altered binding to the RGT heterodimer or to other regulatory proteins. This expectation was based on C‐terminal truncation mutants (Δ9 and Δ33) in the yeast Mrs6p (REP) protein which are viable and only marginally affected (Bauer et al., 1996; Miaczynska et al., 1997). Among the pathological (causing choroideremia) mutations reported so far in the human REP‐1 protein, none is found in the C‐terminal part beyond amino acid position 590 (Esposito et al., 2011; Lin et al., 2011; Strunnikova et al., 2009; Zhou et al., 2012; and others). This may suggest that if mutations in the C‐terminal part of human REP‐1 exist, they might not manifest with disease symptoms.

The alignment of plant REP sequences indicates conservation of the protein region affected in the *rep‐1* mutant. We speculated that some plant‐specific functions could be compromised in this line, due to hypoprenylation of (a) particular Rab(s), lower abundance, or lower REP affinity. In the milder *S. cerevisiae* mutants prenylation of some Rabs seemed unaffected (for example Ypt1, corresponding to the plant Rab‐D subclass), while others were clearly hypoprenylated (Sec4, corresponding to Rab‐E in plants) (Bialek‐Wyrzykowska et al., 2000). In the invertebrate *C. elegans*, suppression of REP expression by RNA interference gave similar effects; some Rabs (Rab27) and connected processes were strongly affected while others (Rab1 and Rab3) maintained their physiological functions (Tanaka et al., 2008). In CHM models also only some Rabs (Rab27 and Rab35 in particular) are hypoprenylated, while others are not (Kohnke et al., 2013; Storck et al., 2019). Our study suggests that a hierarchy of Rab prenylation may exist in plants as well. Small differences in REP preference for particular Rab family members in plants and animals/yeast may be explained by differences in the priorities for transport processes for motile versus sessile lifestyles, unicellular versus organ‐built organisms, or cell wall‐free versus cell wall‐surrounded cells.

Summarizing, the structural basis of the REP–Rab interaction in animals and plants is not the same. The major RBP in REP is involved in Rab binding in both groups. On the contrary, the interaction of REP and Rab C‐termini, proposed to be crucial for efficient Rab prenylation in the mammalian complex, is probably absent in plants. Together with the lack of the highly conserved arginine engaged in RGTA binding (Hala et al., 2005) and the ability to prenylate non‐Rab substrates in the absence of REP (Shi et al., 2016), this is the third major difference in the geranylgeranylation process of Rabs between plants and animals.

## EXPERIMENTAL PROCEDURES

### Plant and bacterial strains

*Arabidopsis thaliana* ecotype Col‐0 was used as the WT line. Lines mutated in the *REP* gene were SALK_140044C (*rep*‐*1*) and GK_295F01 (*rep*‐*2*); the *rep‐1/rep‐2* line was obtained by crossing *REP/rep‐2* to *rep‐1* pollen. The REP‐GFP‐expressing line was constructed by *Agrobacterium* transformation of the WT (for microscopic observations) or the *rep‐1/rep‐2* line with pGWB551‐REP (for genetic reversion of the *rep‐2* phenotype) by the floral dip method. *UB10:*mCherry‐Rab‐expressing lines, wave25 (Rab‐D1), wave27 (Rab‐E1d), and wave2 (Rab‐F2b), came from the Nottingham Arabidopsis Stock Centre (Geldner et al., 2009) and were used as pollen acceptors in *rep*‐*1* crosses. Genotyping was performed with primers given in Table S2. DNA obtained from the genotyping PCR reactions of each mutant line was sequenced to determine the exact localization of the inserts in the *REP* gene.

*Escherichia coli* strain DH5α was used for cloning and BL21(DE3)pLysS or BL21(DE3)Rosetta for protein overexpression.

### Plant growth conditions

Plants were grown under a 16‐h photoperiod (long‐day conditions) in a greenhouse. Seedlings for microscopic observations were grown on vertical plates with ½ MS medium with 1% sucrose supplemented with vitamins and solidified with 1.2% agar. Seedlings for metabolic labeling were grown in liquid MS medium with 1% sucrose for 4 weeks in long‐day conditions with shaking at 150 rpm on a rotary shaker.

### RT‐qPCR analysis

For gene expression analysis, rosette leaves of genotyped 5‐week‐old plants were collected. RNA was extracted using the GeneJET Plant RNA Purification Mini Kit (Thermo Scientific, Waltham, MA, USA) and digested with the RapidOut DNA Removal Kit (Thermo Scientific), and 0.5 μg was reverse‐transcribed using a RevertAid First Strand cDNA Synthesis Kit (Thermo Scientific). The obtained cDNA was quantified by qPCR using SG qPCR Master Mix (2×) plus ROX Solution (EURx, Gdansk, Poland) and a StepOnePlus Real‐Time PCR System (Applied Biosystems, Waltham, MA, USA). The cDNA was diluted 10× and 10 μl was used in a total reaction volume of 25 μl per well. For analysis of the full‐length *REP* transcript, primers REP‐F5 and REP‐R6 were used, and for analysis of the N‐terminal part of the gene, primers REP‐F3 and REP‐R4 were used (primers are listed in Table S2). The gene encoding PROTEIN PHOSPHATASE 2A SUBUNIT A3 (*PP2A*) was used as an internal reference (primers PP2A‐F and PP2A‐R). The expression of each gene was examined in three biological replicates. The relative expression levels were determined using the 2^−ΔΔCt^ method and normalized to expression in WT plants.

### Plasmid construction

Rab genes were cloned from cDNA (prepared from leaves of WT Col‐0 plants with appropriate primer pairs, Table S2) into the pGEX4T1 vector cut with the *Sma*I restriction enzyme. The REP‐6×His sequence was provided on the pET30a‐REP plasmid by Dr. Michal Hala, Charles University, Prague. The pGWB551‐REP plasmid was obtained by cloning the full‐length *REP* sequence without the stop codon (PCR product from WT Arabidopsis Col‐0 cDNA from leaf) into the pENTR vector (Invitrogen, Waltham, MA, USA) and then recombining into the binary vector pGWB551 (Nakagawa et al., 2007) using clonase (Invitrogen). REPΔC was cloned from the pGWB551‐REP vector with the BP‐rep‐F and BP‐rep‐R primer pair, recombined into the pDONR201 vector using the BP‐clonase reaction, and then recombined into the pET301/CT‐DEST vector using the LR‐clonase reaction. Correct orientation and nucleotide sequence of the products were checked by sequencing.

### Protein overexpression and purification

Plasmids were transformed into *E. coli* BL21(DE3)pLyS cells and induced by 1% lactose overnight at 16°C in LB with appropriate antibiotics. Bacteria were pelleted, sonicated, and centrifuged for 30 min at 30 000 ***g***. Supernatant was subjected to affinity chromatography on Ni‐NTA agarose (Sigma‐Aldrich) or Glutathione‐Sepharose4B (Sigma‐Aldrich, Saint Louis, MO, USA) according to the manufacturer’s protocols. REP‐His was further purified on a HiTrapQ 5 ml column (Pharmacia, Chicago, IL, USA) in a gradient of 100–500 mm NaCl. REP‐His, GST‐Rab‐F2b, GST‐Rab‐D1, and GST‐Rab‐E1d were further purified on a Superdex 75 10/300 GL (GE Healthcare, Chicago, IL, USA). The GST tag was digested from GST‐Rab‐E1d with the use of thrombin (Sigma‐Aldrich, Saint Louis, MO, USA) at room temperature overnight, and Rab‐E1d was collected as the flow‐through from the GST‐Sepharose column. Final protein preparations were dialyzed into reaction buffer (Tris 20 mm pH 7.5, 5 mm MgCl_2_, 100 mm NaCl, 1 mm DTT) and concentrated on Amicon Ultra‐15, MWCO 30 kDa (Millipore, Burlington, MA, USA) or MWCO 10 kDa for untagged Rab‐E1d.

### Protein overlay assay

Purified Rab proteins dialyzed into the reaction buffer were diluted to a concentration of 2 mg ml^−1^ and 10‐μl samples were supplemented with GDP or GTP to a final concentration of 10 mm. After 2 h pre‐incubation at room temperature proteins were serially diluted in a 1:10 or 1:5 ratio in the same buffer and spotted on a nitrocellulose membrane. After drying, the membrane was blocked in 1% BSA for 1 h at room temperature and further incubated in 1% BSA containing 0.8 mg purified REP‐His (or REPΔC‐His) and nucleotide at a concentration of 1 mm for 1 h at room temperature. After extensive washing with PBS, the membrane was incubated with monoclonal mouse anti‐His (1:2500; GenScript, Piscataway Township, NJ, USA) in 1% BSA in PBS for 1 h at room temperature followed by washing with PBS and incubation with HRP‐conjugated anti‐mouse antibody (1:2000) for 1 h at room temperature. After washing, the signal was developed with ECL reagent on Kodak X‐Omatic film. Specificity of the anti‐His antibody against REP‐His and GST‐Rabs was checked on Western blot.

### Analytical size‐exclusion chromatography

Purified proteins, REP‐His (4 mg ml^−1^), REPΔC‐His (6.5 mg ml^−1^), GST‐Rab‐F2b (18 mg ml^−1^), GST‐Rab‐D1 (9 mg ml^−1^), and GST‐Rab‐E1d (19 mg ml^−1^), were diluted to 65 nm in 200 μl buffer (Tris 20 mm pH 7.5, 100 mm NaCl, 10 mm MgCl_2_, 1 mm DTT, 1.5 mm GDP) and incubated for 4 h at room temperature. Next, proteins were injected on a Superdex 75 10/300 GL (GE Healthcare, Chicago, IL, USA) equilibrated in the same buffer without GDP. The column was run at 0.5 ml min^−1^ on an AktaPurifier FPLC system (GE Healthcare) and six fractions of 750 μl were gathered starting from 7 min of the run (void volume). Standards of proteins of known masses were used to equilibrate the column (Bio‐Rad, Hercules, CA, USA). Control experiments were performed with purified GST (12 mg ml^−1^) and Rab‐E1d purified after cleavage of the GST tag (2.1 mg ml^−1^). Samples of all fractions from each run were resolved by 12% SDS‐PAGE and stained with Coomassie BB R‐450.

Samples of purified proteins (REP‐His, GST‐Rab‐D1, GST‐Rab‐F2b, and GST‐Rab‐E1d) or equimolar complexes thereof (after SEC column separation) were further concentrated on Amicon filters as described earlier.

### HDX sequencing

The lists of peptides for Rab and REP proteins were obtained using non‐deuterated samples. Twenty microliters of REP‐His (30 µm concentration) with GST‐Rab‐F2b, GST‐Rab‐D1, or GST‐Rab‐E1d (55 µm concentration) was incubated with 30 µl of buffer (20 mm Tris‐HCl, 100 mm NaCl, 5 mm MgCl_2_, 1 mm DTT pH 7.5 supplemented with 50 mm tris(2‐carboxyethyl)phosphine). The mixtures were acidified with 2 m glycine‐HCl pH 2.5. Each protein sample was subjected to on‐line pepsin digestion using a 2.1 mm × 30 mm immobilized pepsin column (Porozyme, ABI, Foster City, CA, USA) with 0.07% formic acid in water as the mobile phase (200 μl min^−1^ flow rate) at 20°C. The generated peptides were trapped on a VanGuard pre‐column (C18, 2.1 mm × 5 mm; Waters, Milford, MA, USA) at a flow rate of 40 µl min^−1^ of solvent A (0.1% formic acid in MQ water). Subsequently, peptides were resolved using an ACQUITY UPLC BEH C18 reverse phase column (1.0 mm x 10 mm, Waters) with a 6–40% gradient of acetonitrile in 0.1% formic acid at a flow rate of 40 µl min^−1^ at 0.5°C. Following the chromatographic separation, the peptides were analyzed using a Synapt G2 HDMS mass spectrometer (Waters, Milford, MA, USA), calibrated with sodium formate clusters. Leucine enkephalin was used as a lock mass (200 pg μl^−1^ leucine enkephalin in 50:50 H_2_O:ACN + 0.1% FA). For protein identification, mass spectra were acquired in MSE mode over the *m*/*z* range of 50–2000. The spectrometer parameters were as follows: ESI positive mode, capillary voltage 3 kV, sampling cone voltage 35 V, extraction cone voltage 3 V, source temperature 80°C, desolvation temperature 175°C, and desolvation gas flow 800 L h^−1^. Peptides were identified using ProteinLynx Global Server software (Waters, Milford, MA, USA). The lists of peptides were further filtered in DynamX 3.0 software (Waters) with criteria: minimum intensity, 1000; minimum products per amino acid, 0.3.

### Hydrogen–deuterium exchange

The H‐D exchange reactions were performed by mixing 5 μl of each protein with 45 μl of reaction buffer containing 20 mm Tris‐DCl, 100 mm NaCl, 5 mm MgCl_2_, 1 mm GDP pH 7.5 prepared with D_2_O (99.8%; Cambridge Isotope Laboratories, Tewksbury, MA, USA) and pH (uncorrected meter reading)‐adjusted using DCl (Sigma‐Aldrich). The exchange reactions were performed for specific time points (10 sec, 1 min, and 60 min). Then, the exchange was quenched with the addition of 10 µl of 2 m glycine pH 2.5 prepared in D_2_O cooled on ice. Two control experiments were conducted to assess the minimum and maximum H‐D exchange levels. For minimal exchange analysis (M_min_), 10 µl of a quench buffer was mixed with 45 µl of D_2_O reaction buffer prior to the addition of 5 µl of protein stocks. To obtain the maximal exchange level (M_max_), the deuteration reaction was conducted over 2 days and then quenched on ice.

HDX‐MS sample analysis was performed as described for non‐deuterated samples, but additionally deuterated peptides were separated by MS operated in ion mobility mode. All raw files were processed and analyzed in DynamX 3.0 software. The percentage of deuteration [D%] was calculated in Excel from exported DynamX 3.0 data, based on the following formula, which takes into account the minimal and maximal exchange of a given peptide:

$$D\;\left[\%\right]=\frac{\left(M‐M_{\min}\right)}{\left(M_{\max}‐M_{\min}\right)}*100\%,$$

where M is the centroid mass of a given peptide after deuterium uptake, M_min_ is the centroid mass of a peptide with minimal exchange, and M_max_ is the centroid mass of a peptide with a maximal exchange. The experiments were performed in triplicate.

The difference in exchange between two states (apo state and in a complex) was obtained by subtracting the percentage of deuteration measured for a given peptide at a specific experimental setup. Errors for this difference were calculated as the square root of the sum of variances of the subtracted deuteration values. Only peptides with statistically significant reproducibility were plotted (Moller et al., 2019). Final figures were plotted using OriginPro 9.0 (OriginLab, Northampton, MA, USA) software.

### Preparation of plant lysates and Western blots

Leaves of 5‐week‐old plants grown under long‐day conditions were homogenized in liquid nitrogen with a mortar and pestle in buffer containing 0.3 m sucrose, 50 mm NaCl, 25 mm HEPES pH 7.2, 5 mm MgCl_2_, 0.1 mm PMSF, and protease inhibitor cocktail (Complete Mini, Roche, Bazylea, Switzerland). The homogenate was centrifuged at 10 000 ***g*** for 30 min. Mouse anti‐AtREP antibody (a gift of Dr. Michal Hala, Charles University, Prague) was used at 1:1000. Mouse anti‐GFP antibody (pAB290; Abcam, Cambridge, UK) was used at a 1:1000 dilution. HRP‐conjugated goat anti‐mouse IgG (Sigma‐Aldrich) was used as secondary antibody at a 1:1000 dilution. Detection of the signal was performed with the SuperSignal WestPico chemiluminescence kit (Pierce, Waltham, MA, USA) on Kodak X‐Omat AR5 film (Sigma‐Aldrich).

### [^3^H]geranylgeranylation

Chemical synthesis was performed following (Keenan and Kruczek, 1975). Obtained [^3^H]geranylgeranyl alcohol, specific activity 2.5 Ci mol^−1^, was dissolved in hexane at a concentration of 3.7 × 10^7^ dpm μl^−1^. *In vivo* labeling and extract preparation were performed as described in (Gutkowska et al., 2004) and proteins were separated by 15% SDS‐PAGE. Fractions corresponding to 20–30 kDa were cut from the gel, solubilized in 5.5% H_2_O_2_ at 65°C for 24 h, and measured for radioactivity in a Tri‐Carb 2910TR liquid scintillation counter (Perkin Elmer, Waltham, MA, USA) with Insta‐Gel Plus scintillation liquid (Packard, Waltham, MA, USA). Bands of the same molecular mass from the same gel but containing non‐labeled samples were treated as control. For gel autoradiography, SDS‐PAGE gels were soaked in salicylic acid as described in (Hala et al., 2010), dried under vacuum, and exposed on Kodak X‐Omat AR5 film at −70°C for 1 month.

### Pollen grain staining and pollen germination

Mature anthers just before dehiscence were fixed and stained with Alexander stain according to (Lalanne et al., 2004). Anthers were observed under an inverted TE2000 microscope (Nikon Instruments, Amsterdam, The Netherlands). Image acquisition was performed with the use of a color camera and NIS‐Elements software (Nikon, Amsterdam, The Netherlands).

*In vitro* pollen germination was conducted as described in (Boavida and McCormick, 2007). Microscopic observations were performed on an E800 Eclipse Nikon microscope equipped with a CCD Hamamatsu monochromatic camera. The length of pollen tubes was measured using ImageJ programme. Anthers coming from flowers at anthesis were soaked in 1 µg ml^−1^ of DAPI stain in water for 24 h at 4°C. DAPI solution was decanted and anthers were washed in fresh water. Observations were conducted on an E800 Eclipse Nikon fluorescent microscope (Nikon Instruments) and recorded on a CCD Hamamatsu monochromatic camera. An excitation filter at 340–380 nm was used.

### Confocal microscopy

Localization of mCherry‐Rab fusion proteins in *rep*‐*1* and WT backgrounds and of REP‐GFP fusion proteins in the WT background was observed in cotyledon epidermis, root epidermis, root hairs, and the root meristematic zone of 5–7‐day‐old seedlings grown under long‐day conditions on vertical ½ MS plates with 1% saccharose by confocal laser scanning microscopy. Experiments were performed on at least six plants coming from three independent plant cultivations. Fluorescence imaging was performed on a Nikon C1 confocal system built on TE2000E and equipped with a 40× PlanFluor and 60× Plan‐Apochromat oil immersion objective (Nikon Instruments B.V. Europe, Amsterdam, The Netherlands). GFP was excited with a Sapphire 488 nm laser (Coherent, Santa Clara, CA, USA) and observed using the 515/530 nm emission filter. mCherry was excited with a 543 nm HeNe laser and detected using the 605/675 nm emission filter. Images were collected in single plane or z‐stack mode at a 1 µm‐focus interval. Microscopy pictures were prepared in ImageJ.

### Transmission electron microscopy

Leaf sections (2 × 2 mm) from WT and *rep*‐*1* plants were vacuum fixed in 2.5% glutaraldehyde in 0.1 m cacodylate buffer pH 7.2 for 2 h and post‐fixed in 1% osmium tetroxide for 2 h. After washing in cacodylate buffer the sections were dehydrated through an ethanol series, embedded in Epon‐Spurr resin (Sigma‐Aldrich), and polymerized for 48 h at 60°C. WT and *rep*‐*1* mutant root tips (5 days old) and slices of stems (6 weeks old) were fixed in 5% paraformaldehyde/0.5% glutaraldehyde in 1× PBS for 4 h at room temperature followed by the procedure described above. The sections were stained with uranyl acetate and lead citrate and observed using a JEM‐1200 EX electron microscope (JEOL, Mushashino, Japan).

### Statistical analysis

Statistical analysis was performed with GraphPad5 (GraphPad, San Diego, CA, USA) software. For analysis of trait inheritance, the Fisher exact test or the χ^2^ test was performed against appropriate H_0_ hypotheses, as described in Tables 1 and S1. For analysis of pollen tube length, pollen tubes were measured using ImageJ and the mean ± SD was plotted. The significance of the difference was calculated by the Student *t*‐test against the H_0_ hypothesis that the lengths are equal. For quantification of cell wall width and MVB diameter, ImageJ was used and the median value was plotted. Boxes show 50 percentiles of the data points and the whiskers mark the 95% confidence interval for the calculated median.

### Alignments

Protein sequences were retrieved from the EMBL server by repeated FASTA searches with known REP or Rab sequences from plants and other organisms. Protein alignments were performed using MUSCLE (http://www.ebi.ac.uk/Tools/msa/muscle/). Alignments were replicated 100 times using the bootstrap method in the SEQBOOT algorithm. The alignments were edited in Jalview (http://www.jalview.org) to remove the gaps.

## AUTHORS CONTRIBUTION

MG, LS, MH‐S, and ES conceived the work plan, MG, MK‐D, MH‐S, MMP, ADL, ML, MW, LS, and AW performed the experiments, MG, MK‐D, MP, and MH‐S analyzed the data, MG, MK‐D, and MH‐S wrote the manuscript, and MG, ES, MD, and LS supervised the work and provided financial support.

## CONFLICT OF INTEREST

The authors declare no conflicts of interest.

## Supporting information


**Figure S1**. Control experiments for the *in vitro* interaction of REP and Rabs.
**Figure S2**. Peptide libraries for the HDX‐MS experiments.
**Figure S3**. GST protein control in the HDX‐MS experiments.
**Figure S4**. Alignment of REP protein sequences.
**Figure S5**. Secondary structure prediction for the REP protein.
**Figure S6**. Structural models of Arabidopsis REP and selected Rab proteins.
**Figure S7**. Phenotypes of *rep*‐*1* and *rep*‐*2* mutants and revertant plants.
**Figure S8**. Reversion of the pollen phenotype of the *rep*‐*1/rep*‐*2* line.
**Figure S9**. Ultrastructure of the *rep*‐*1* mutant.
**Figure S10**. Protein models of full‐length REP and C‐terminally truncated versions.
**Table S1**. Genetic segregation of the progeny of *rep‐1/rep‐2 35S:REP‐GFP* hemizygous plants.
**Table S2**. List of primers.

## Data Availability

All data and supplementary data are available online from the website of *The Plant Journal*.
