# Supplementary material for: Impact of C‐terminal truncations in the Arabidopsis Rab escort protein (REP) on REP–Rab interaction and plant fertility
Source: Plant J. 2021 Oct 16;108(5):1400–21. doi: 10.1111/tpj.15519 (PMC9293207; doi:10.1111/tpj.15519)
Supplement: Supplementary file 1 — Figure S1. Control experiments for the in vitro interaction of REP and Rabs. Figure S2. Peptide libraries for the HDX‐MS experiments. Figure S3. GST protein control in the HDX‐MS experiments. Figure S4. Alignment of REP protein sequences. Figure S5. Secondary structure prediction for the REP protein. Figure S6. Structural models of Arabidopsis REP and selected Rab proteins. Figure S7. Phenotypes of rep‐1 and rep‐2 mutants and revertant plants. Figure S8. Reversion of the pollen phenotype of the rep‐1/rep‐2 line. Figure S9. Ultrastructure of the rep‐1 mutant. Figure S10. Protein models of full‐length REP and C‐terminally truncated versions. Table S1. Genetic segregation of the progeny of rep‐1/rep‐2 35S:REP‐GFP hemizygous plants. Table S2. List of primers. [file TPJ-108-1400-s001.pdf]

SUPPLEMENTARY MATERIAL

Impact of C-terminal truncations in the *Arabidopsis* Rab Escort Protein (*REP*) on REP-Rab interaction and plant fertility

Fig. S1

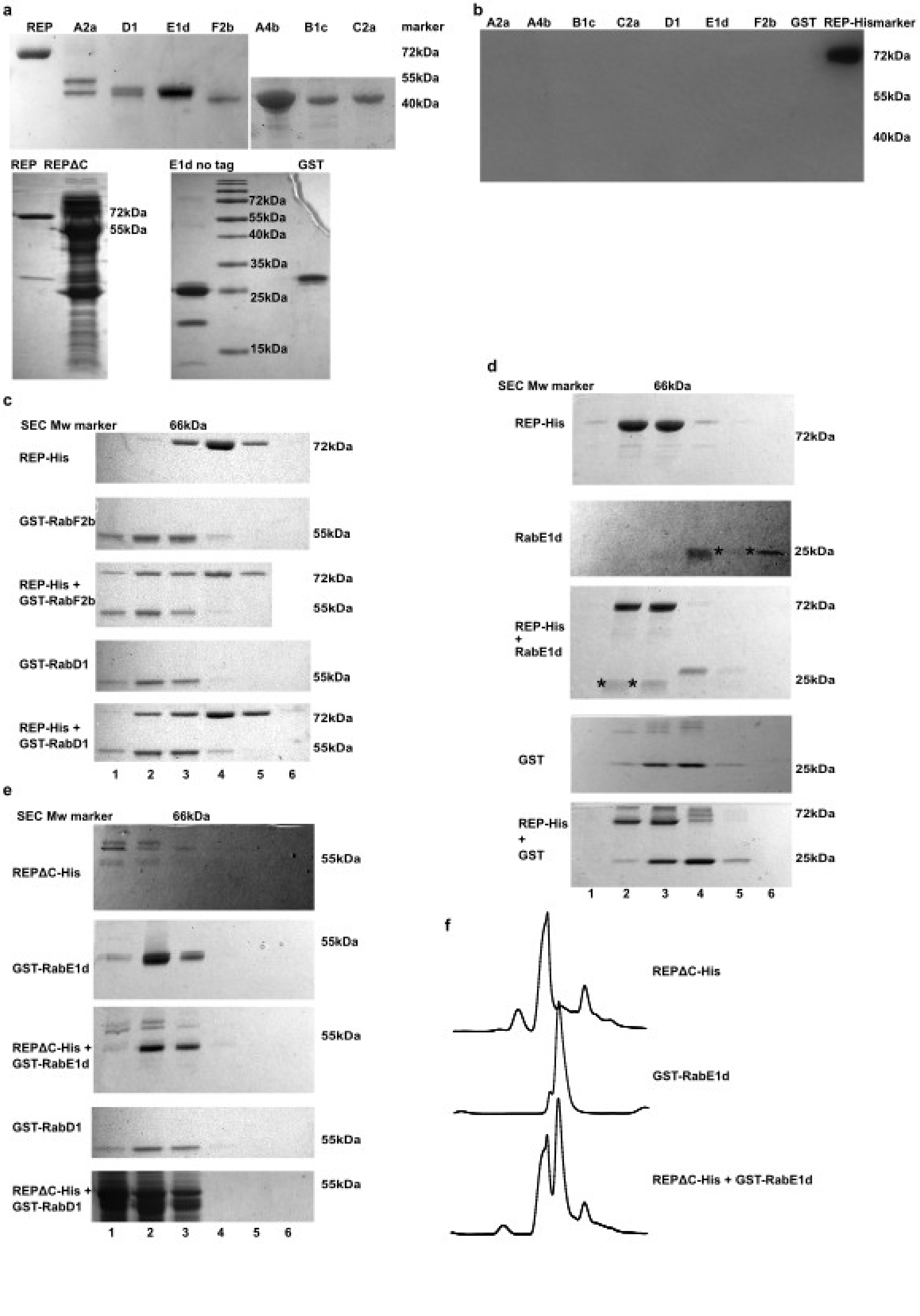

Fig. S1 Control experiments for the *in vitro* interaction of REP-His with GST-Rabs

a) Purity of proteins used for *in vitro* experiments. Equal amounts of the protein preparations were resolved on SDS-PAGE and stained with CBB R-450. **b)** None of the GST-Rab proteins used for the blot overlay assays is recognized by the anti-His antibody, Western blot, equal amounts of the proteins were loaded. **c)** GST-RabF2b and GST-RabD1 bind to REP-His. SDS-PAGE gels from the SEC runs shown on Fig. 1e. **d)** RabE1d cleaved from its GST tag interacts with REP-His (upper panels). Asterisks denote RabE1d bands, note the unspecific band of slightly slower electrophoretic mobility that does not bind to REP. The respective chromatogram is shown on Fig. 1f. The GST alone does not bind to REP-His (lower panels). **e,f)** REPΔC-His migrates with the void volume of the column, probably due to oligomerization, but binds GST-RabD1 (see also chromatogram Fig. 1g) and GST-RabE1d (shown on chromatogram Fig. S1f). The same control for GST-RabE1d interaction with REP-His and REPΔC-His was used (SDS-PAGE gels shown on Fig. 1d and Fig. S1e) and the same control for GST-RabD1 interaction with REP-His and REPΔC-His was used (SDS-PAGE gels shown on Fig. S1c and Fig. S1e).

Fig. S2

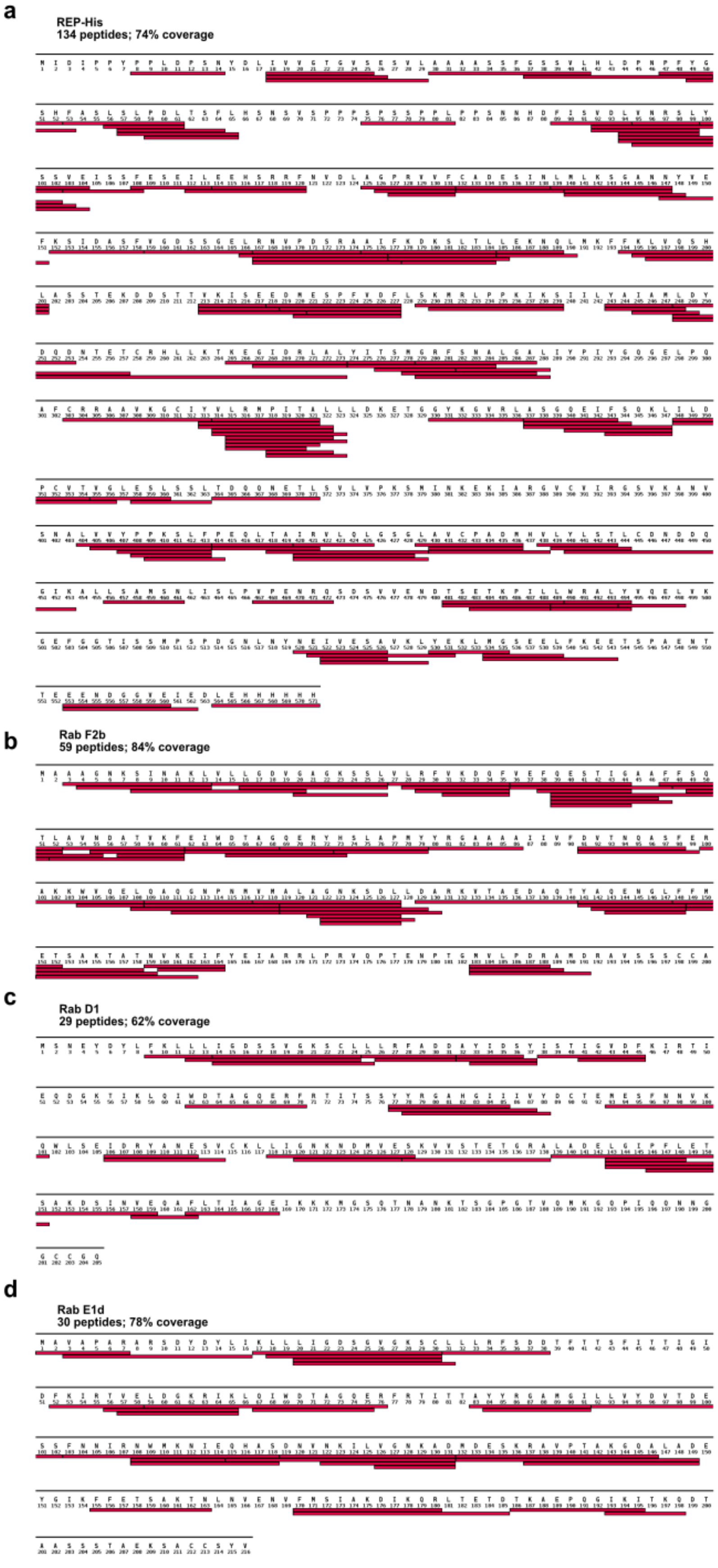
a
REP-His
134 peptides; 74% coverage
b
Rab F2b
59 peptides; 84% coverage
c
Rab D1
29 peptides; 62% coverage
d
Rab E1d
30 peptides; 78% coverage

Fig. S2 Peptide libraries for proteins used for the HDX-MS experiments

a) REP-His, **b**) RabF2b, **c**) RabD1 and **d**) RabE1d proteins were sequenced by the means of MS after pepsin cleavage. Peptide coverage maps were created by the program Draw Map. The maps were used as libraries for peptides derived from the HDX-MS experiments.

Fig. S3

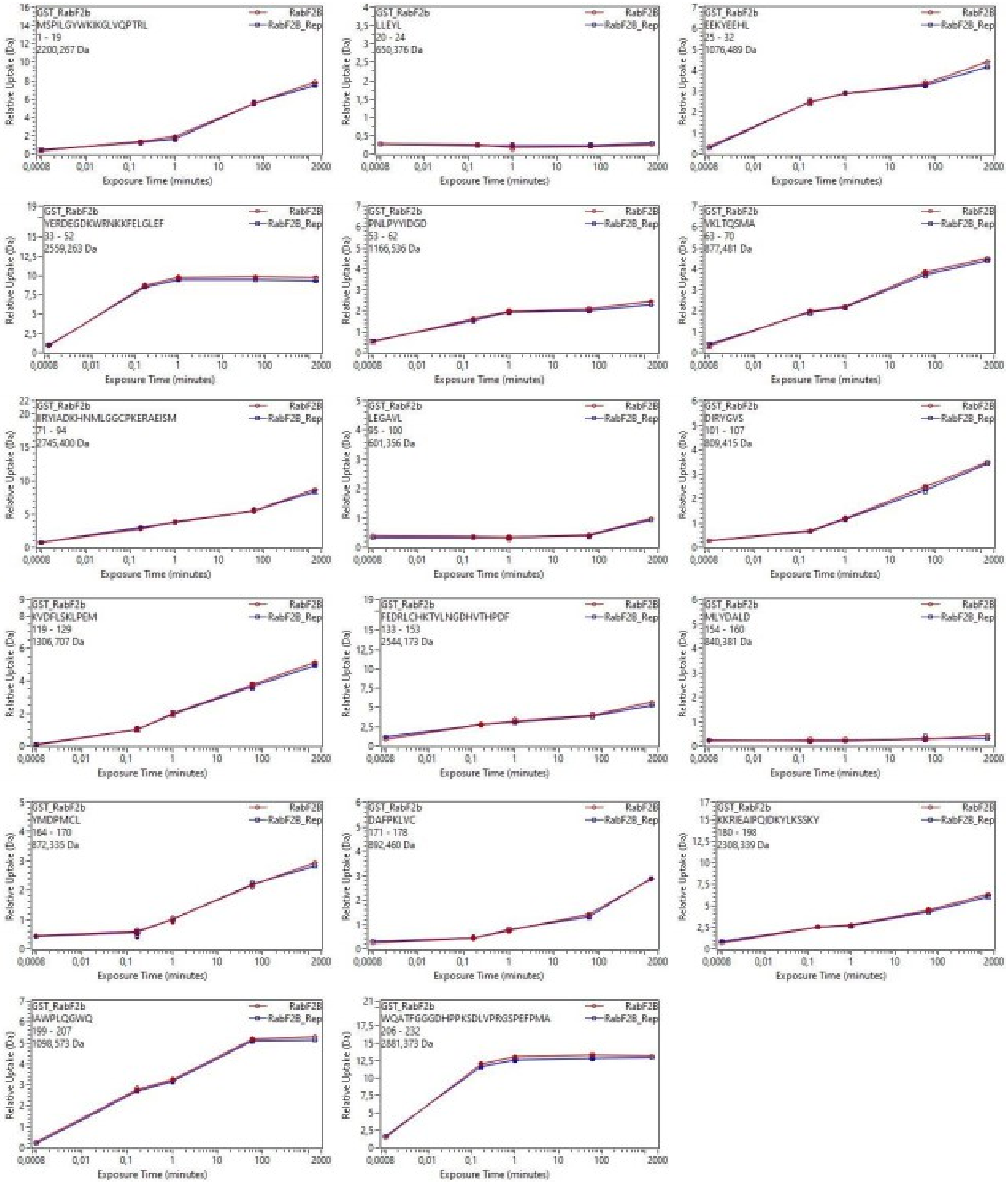

Fig. S3 The GST protein does not show any changes in HDX upon REP-His binding

Deuterium uptake by selected GST peptides, covering the whole peptide sequence of the protein, do not show any changes in H-D exchange upon REP binding [Da]. Each point represents the mean centroid mass of a peptide coming from protein or complex incubated in D_2O, +/- SD. Sequences of consecutive GST peptides are denoted on the graphs. Results come from the experiment in which the interaction of GST-RabF2b with REP-His was studied.

Fig. S4

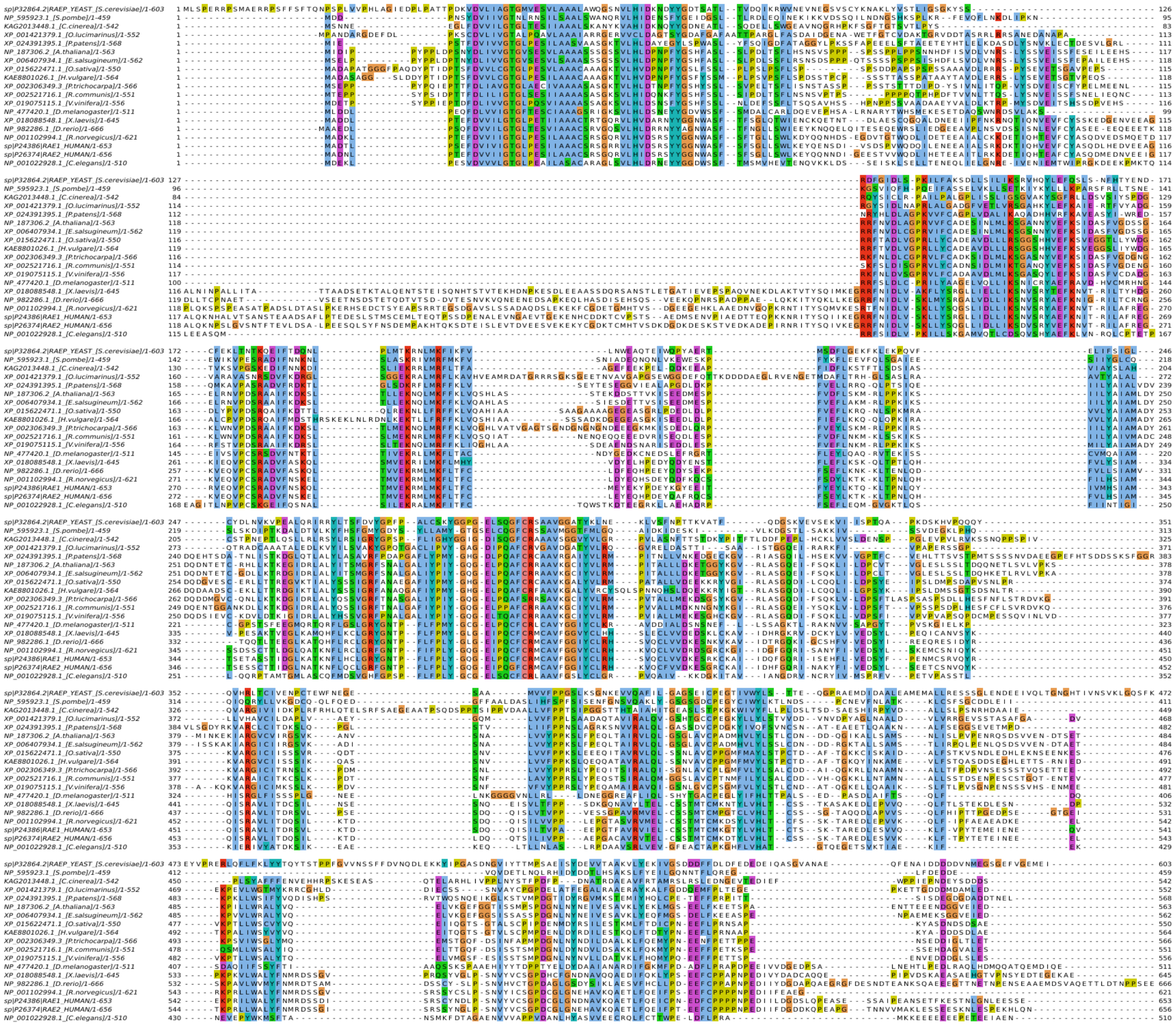

Fig. S4 Alignment of REP protein sequences from organisms representing different phylogenetic groups.

Full length amino acid sequences of REP proteins (or putative REP) from organisms representing different kingdoms of life were compared. Regions of high similarity as well as highly divergent regions are visible. In particular note the low conservation of the C-terminus. Protein alignments were calculated using MUSCLE (http://www.ebi.ac.uk/Tools/msa/muscle/) and edited in Jalview (http://www.jalview.org) to remove gaps. Amino acids are colored according to their chemical properties.

Fig. S5

a

SOPMA

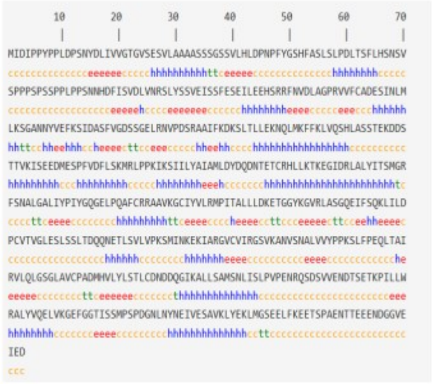

b

NetSurfP 2.0

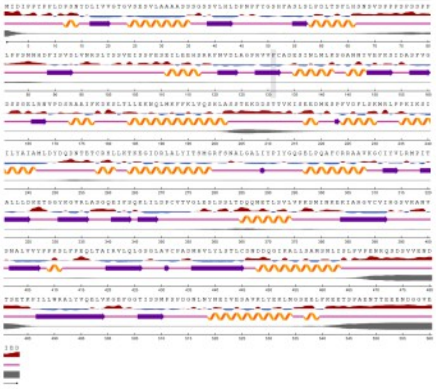

c

JPred4

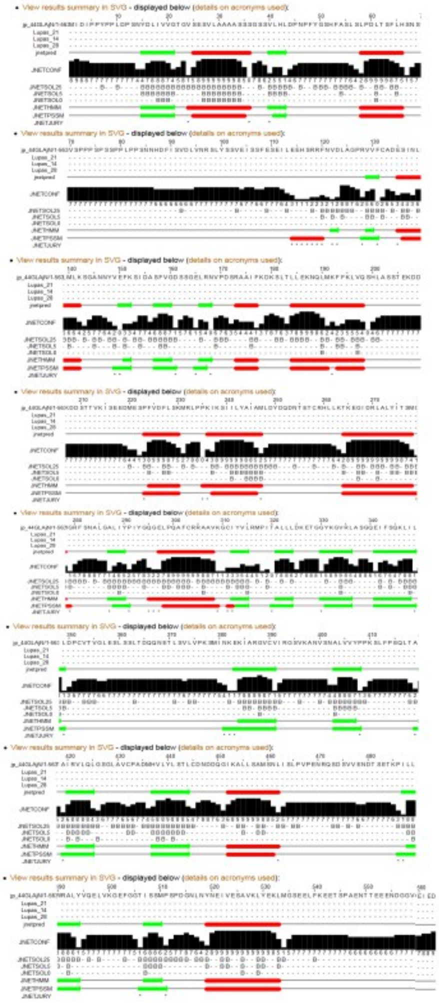

d

Gor4

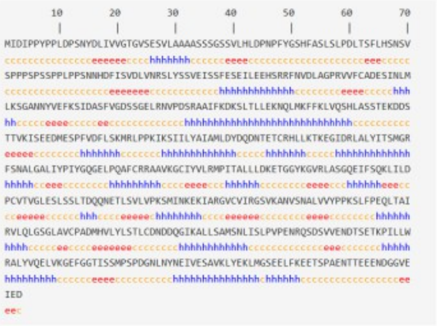

e

GlobPlot

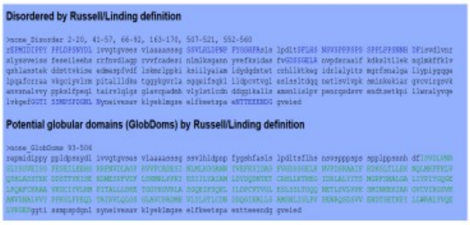

f

DisEMBL

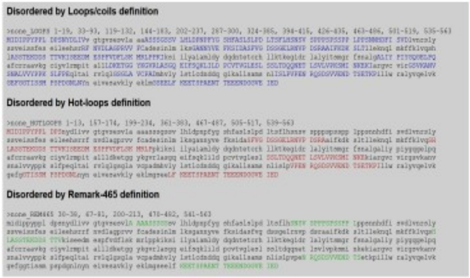

g

CFSSP

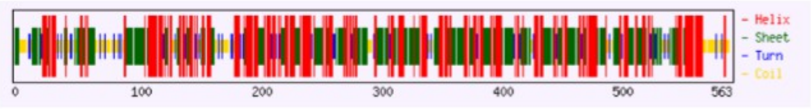

Fig. S5 Secondary structure prediction for the amino acid sequence of the *Arabidopsis thaliana* REP protein

Secondary structure prediction for the REP amino acid sequence was performed based on different algorithms. Note that all predictions are consistent in predicting a C-terminal helix, spanning amino acids 518-535, followed by an unstructured region.

Fig. S6

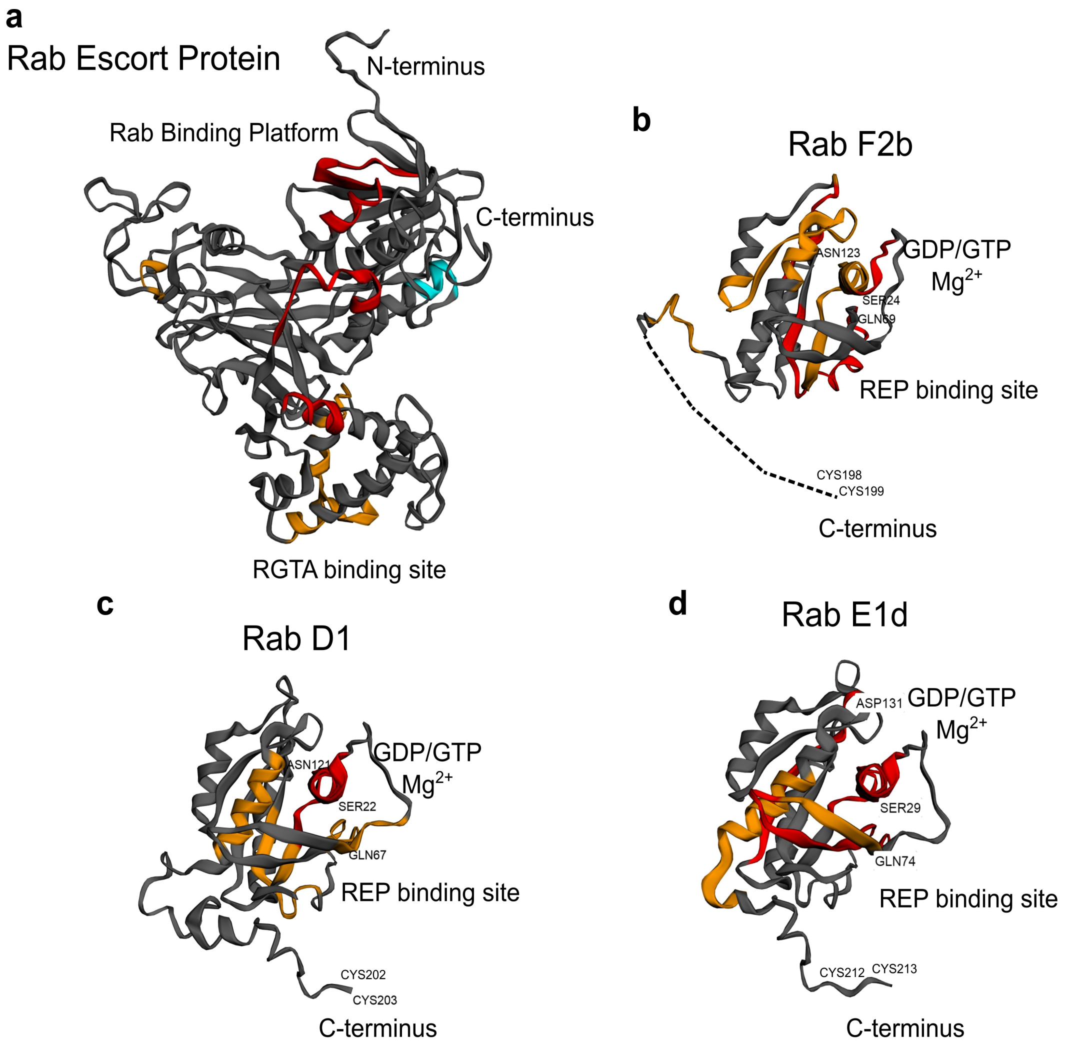

Fig. S6 Structural models of Arabidopsis REP and selected Rab proteins

Structural models of a) REP, b) RabF2b, c) RabD1 and d) RabE1d were built using the PHYRE2 server basing on Hidden-Markov-Model calculations. Templates for modeling were: the crystal structures of mammalian REP and yeast GDI for REP, and for Rabs crystal structures of Rab proteins from different organisms, but from corresponding families. Amino acid stretches corresponding to peptides with high HDX protection are colored in red, of lower but significant HDX protection in orange, and showing higher HDX exchange in blue. Positions of N- and C-termini of proteins are marked as well as amino acids engaged in Mg^{2+} and guanine nucleotide binding and the prenylatable cysteines.

Fig. S7

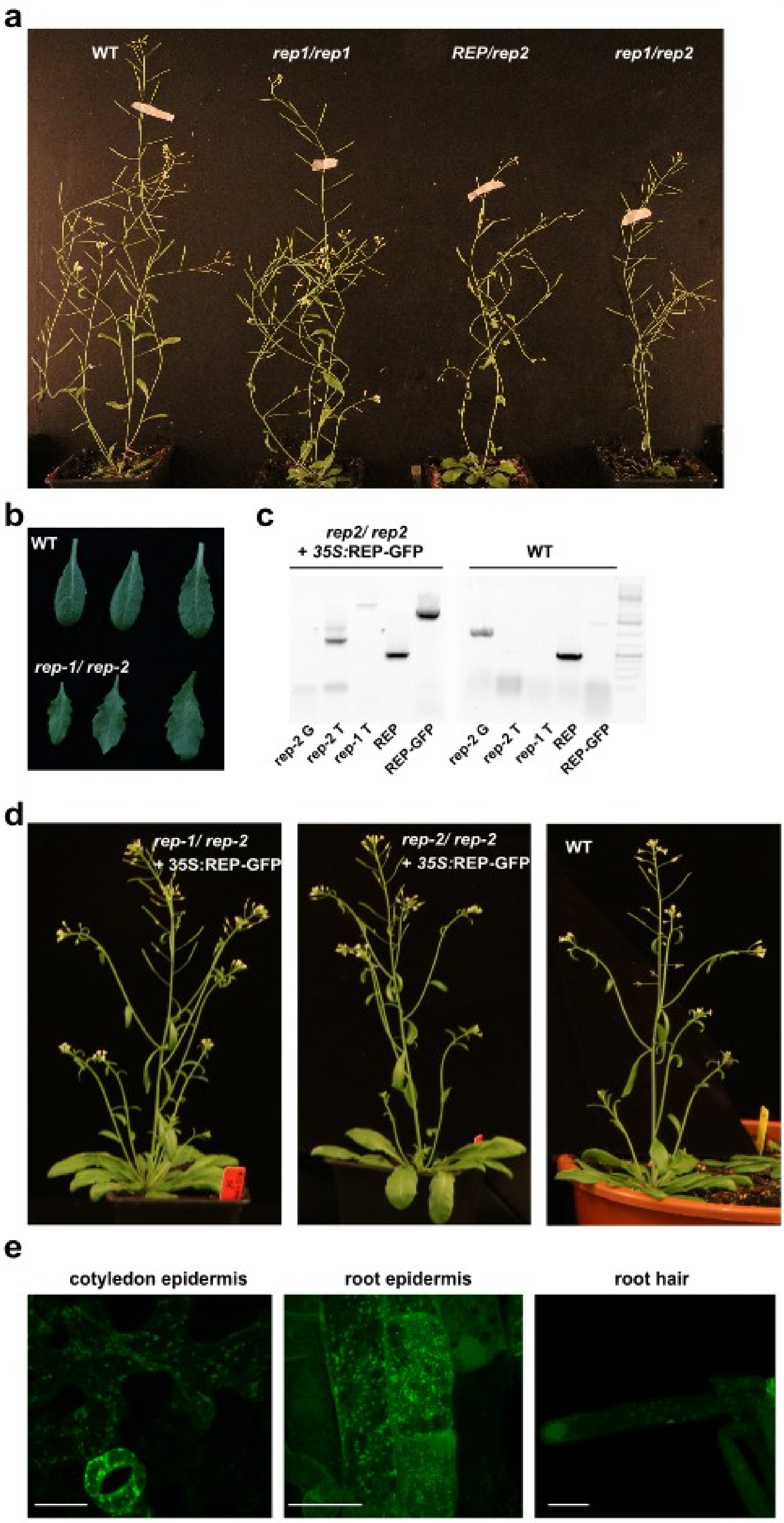
a
WT
rep1/rep1
REP/rep2
rep1/rep2
b
WT
rep-1/ rep-2
c
rep2/ rep2
+ 35S:REP-GFP
WT
rep-2 G
rep-2 T
rep-1 T
REP
REP-GFP
rep-2 G
rep-2 T
rep-1 T
REP
REP-GFP
d
rep-1/ rep-2
+ 35S:REP-GFP
rep-2/ rep-2
+ 35S:REP-GFP
WT
e
cotyledon epidermis
root epidermis
root hair

Fig. S7 Phenotypes of *rep-1* and *rep-2* mutants and revertant plants in comparison to WT plants

a) 6-week old plants grown in soil in long day conditions, note slightly retarded growth of the *rep-1*/*rep-2* and *REP/rep-*2 lines and normal growth of the *rep-1/rep-1* line. **b)** Leaves of the *rep-1/rep-2* plants are more serrated than those of WT plants. **c)** PCR analysis of the genotype of a *rep-2/rep-*2 revertant plant expressing *35S:REP-GFP* – the progeny of a *rep-1*/*rep-2* plant transformed with the pGWB551:REP vector. PCR analysis was run using genomic DNA as templates, with primer pairs specific for: the WT genomic sequence flanking the T-DNA insertion in the *rep-2* allele (rep-2 G), the T-DNA insertion in the *rep-2* allele (rep-2 T), the T-DNA insertion in the *rep-1* allele (rep-1 T), a region of the *REP* gene upstream of both T-DNA insertions (REP), a region spanning the *REP-GFP* junction (REP-GFP). **d)** The viability of *rep-2*/*rep-2* plants is rescued by the *35S:REP-GFP* construct. Shown are 7-week-old plants of the genotypes *rep-1*/*rep-2 35S:REP-GFP* and *rep-2/rep-2 35S:REP-GFP*, both derived as progeny from a *rep-1/rep-2* parent transformed with pGWB551-REP, compared with a WT plant. **e)** CSLM images showing the intracellular localization of REP-GFP in cotyledon epidermal pavement cells, root epidermal cells, and root hairs of WT Col-0 plants. Bars represent 20 μm for cotyledon epidermis image and 10 μm for the other two images. Note the cytoplasmic and vesicle-like structures.

Fig. S8

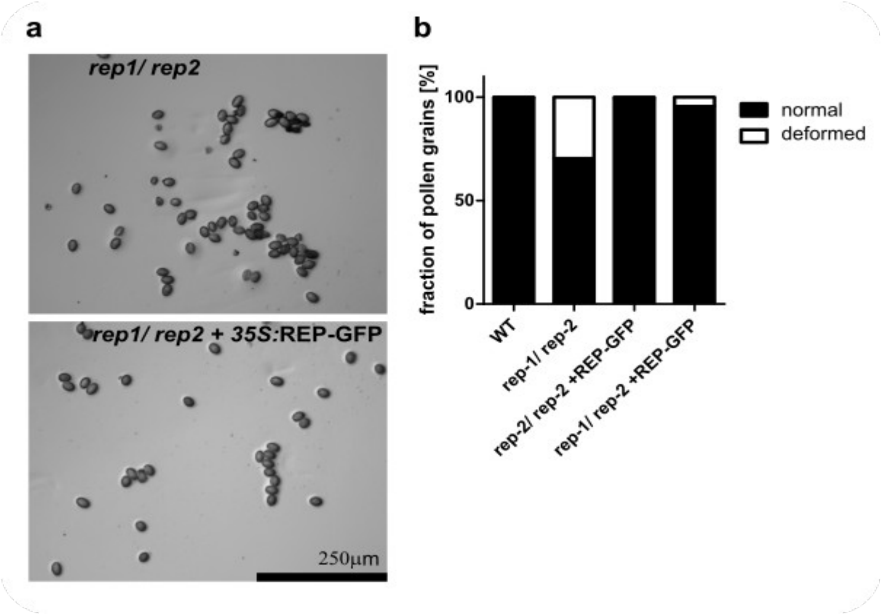

Fig. S8 Reversion of the pollen morphology phenotype of the *rep-1/rep-2* mutant by REP-GFP expression

a) Mature pollen grains from plants of the indicated genotypes were observed by DIC microscopy. Hardly any deformed pollen grains could be observed for the *rep-1/rep-2 35S:REP-GFP* hemizygous line. Scale bar represent 250 µm for both images. **b**) Pollen morphology was quantified for the lines observed in **a** as well as for the *rep-2/rep-2 35S:REP-GFP* hemizygous revertant line. At least 300 pollen grains were counted for each line.

Fig. S9

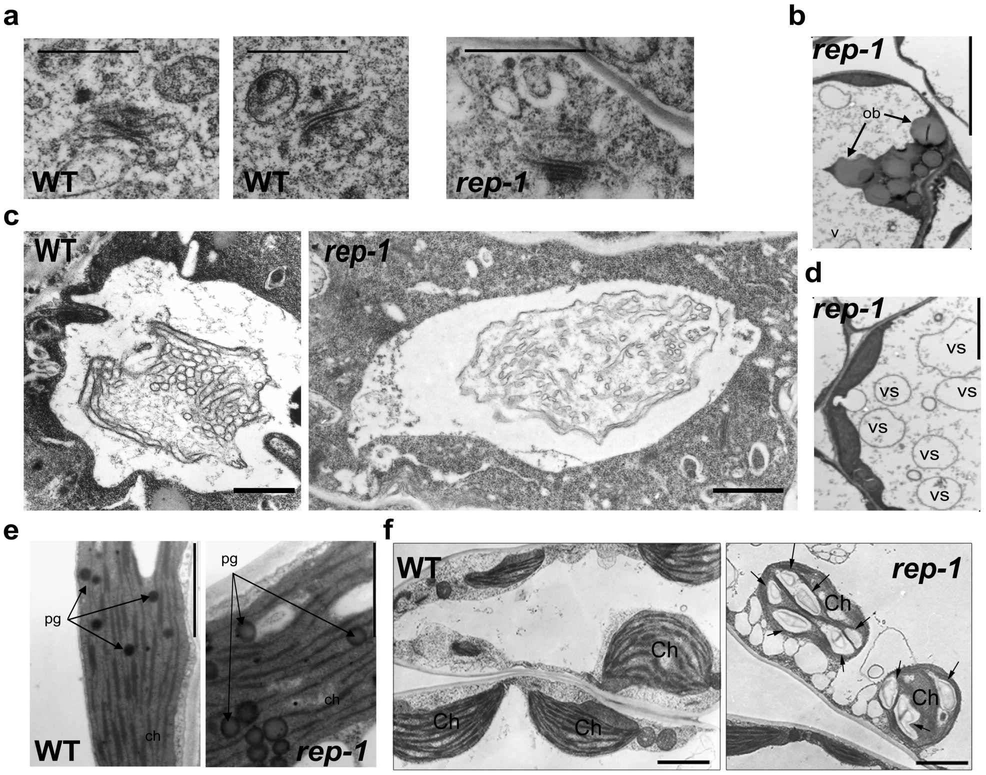

Fig. S9 Ultrastructure of *rep-1* mutant plant cells

Transmission electron microscopy of Arabidopsis thaliana *rep-1/rep-1* mutant tissues does not reveal significant changes in most vesicular structures in comparison to WT. **a)** Golgi structures in root parenchymal cells are similar in size and complexity, **c)** storage vacuoles in stem parenchymal cells are similar in size and shape, **b, d)** cytoplasmic clusters of oil bodies **(b)** and vesicles **(d)** were often observed in *rep-1* root parenchymal cells, but were difficult to quantify, **e, f)** intrachloroplastic plastoglobules **(e)** and starch granules **(f)** were characteristically enlarged in *rep-1* leaf and stem mesophyll cells, especially if the material was gathered at the end of the dark period of the day cycle. Bars 1 µm for b, d pictures and 2 µm for a, c, e, f pictures. Images are representative for at least 10 pictures, each showing several structures of the kind.

Fig. S10

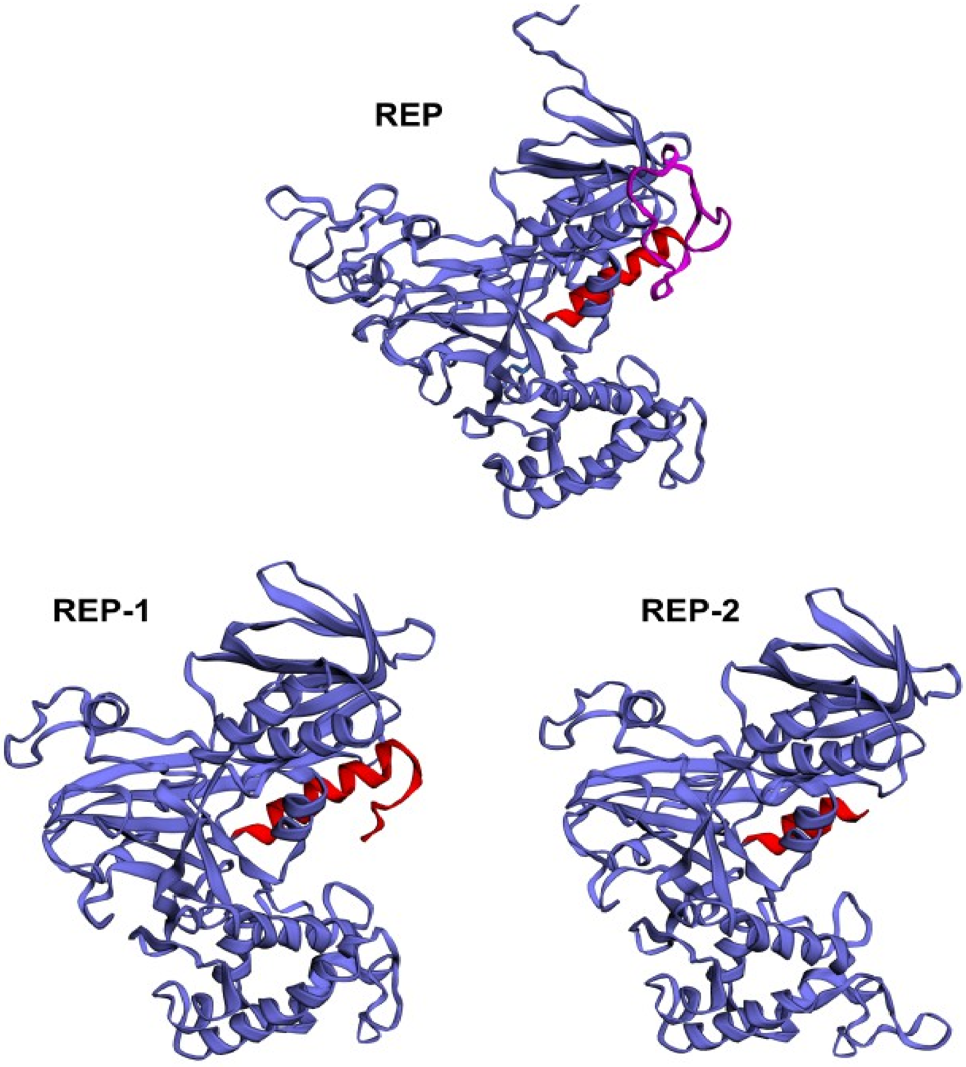

Fig. S10 Protein models of full length plant REP, a C-terminally truncated version corresponding to the protein present in the *rep-1* mutant, and a C-terminally truncated version corresponding to the version present in the *rep-2* mutant.

Structural models of the proteins REP, REP-1 and REP-2 were built using the PHYRE^2 server based on Hidden-Markov-Model calculations. Templates for modeling were the crystal structures of mammalian REP and yeast GDI. Amino acid stretches corresponding to the unstructured C-terminus are colored in magenta and the predicted C-terminal α-helix is in red. In REP-1 only the unstructured C-terminus is lacking, in REP-2 the insertion disrupts the secondary structure of the α-helix and possibly its tertiary interaction with the vast β-sheet of the larger REP domain. Note also that the modeling predicts some changes of geometry of distant tertiary structure elements, in particular of the putative RGTA binding loops of the smaller REP domain and the large loop-helix-loop structure at the opposite side of the REP molecule.

Table S1.

**cross**	**expected ratio** ***rep-1/rep-1 : rep1/rep-2 : rep-2/rep-2***	**observed** ***rep-1/rep-1 : rep1/rep-2 : rep-2/rep-2***	**number of observations**	**p-value**	**p**
rep-1/rep-2 (self)	1:1:0	79:73:0	152	0.8186	NS
rep-1 rep-2 35S:REP-GFP hemizygous (self)	1:1.5:0.5	33:91:16	140	0.04	*
	**expected ratio WT :** ***35S:REP-GFP***	**observed WT :** ***35S:REP-GFP***	**number of observations**	**p-value**	**p**
rep-1/rep-2 35S:REP-GFP hemizygous (self)	1:5	19:121	140	0.616	NS

Table S1. Genetic analysis of mutated *REP* alleles transmission in the *rep-1/rep-2 35S:REP-GFP* hemizygous revertant

rep1/rep-2 or *rep-1/rep-2 35S:REP-GFP* (hemizygous for the *35S:REP-GFP* allele) were left for self pollination. Progeny was grown on 1/2 MS agar plates for 3 weeks, DNA was isolated from whole seedlings and genotyped by PCR with appropriate primer pairs (Table S2). Results were analyzed by $\chi 2$ test or Fisher exact test against the following H_0 hypotheses: in case of *rep-1/rep-2* self-cross that *rep-2* pollen is sterile (as derived from previous crosess analyzed in Table 1); in case of the *rep-1/rep-2 35S:REP-GFP* (hemizygous for the *35S:REP-GFP* allele) that the REP-GFP expression from *35S* promoter enables full reversion of the *rep-2* pollen sterility; in case of the REP-GFP construct in *rep-1/rep-2 35S:REP-GFP* (hemizygous for the *35S:REP-GFP* allele), that the segregation is Mendelian. * marks $p<0.05$, NS marks non-significant result. Note that the p-value obtained for the data *rep-1/rep-2 35S:REP-GFP* (hemizygous for the *35S:REP-GFP* allele) that the REP-GFP expression from *35S* promoter enables full reversion of the *rep-2* pollen sterility is close to to rejection border ($p<0.05$) and the hypothesis does not take into account that the fitness of the *rep-1* pollen is lower than 100%.

Table S2. List of primers used in this work

	Primer name	Primer sequence 5'-3'	Primer purpose
1	RabA2aR	CGCAAGCCTTGAAATAAAGTCTCA	cloning *RabA2a*
2	RabA2aL	GATGGCGAGAAGACCGGACGAAG	
3	RabB1bR	GGCATACACGTGTATTGTCAAACCG	cloning *RabB1b*
4	RabB1bL	CATGTCTTATGCTTATCTCTTCAAG	
5	RabC2aR	ACACACAAAAGAGGCAACCAC	cloning *RabC2a*
6	RabC2aL	CATGGGATCTTCTTCTGGGCA	
7	RabD1R	TCAAGACACAGCGACATGGA	cloning *RabD1*
8	RabD1L	CATGAGCAACGAGTACGATTATCTG	
9	RabE1dR	GTGCAGGCGAAAAAGAGCAAT	cloning *RabE1d*
10	RabE1dL	GATGGCGGTTGCGCCG	
11	RabF2bR	GACGCAAACACAGAAGTCCA	cloning *RabF2b*
12	RabF2bL	CATGGCTGCAGCTGGAAACAAGA	
13	RabA4bR	TCAAGAAGAAGTACAACAAGTGCTGGTC	cloning *RabA4b*
14	RabA4bL	CACCATGGCCGGAGGAGGC	
15	Lbb1.3	ATTTTGCCGATTTCGGAAC	genotyping SALK lines
16	RP140044	TCGGGTTCTGCAGCTTGGTAG	genotyping *rep-1* line
17	LP140044	TGGACTTGGCCATGAAAATCA	
18	Go8409	ATATTGACCATCATACTCATT	genotyping GK lines
19	RPG295F01	TGGTAGTGGTTTAGCGGTTTG	genotyping *rep-2* line
20	LPG295F01	AACGATTATGGTTGTGATCGG	
21	REPgwL	CACCATGATCGACATCCCTCCTTAC	cloning *REP*
22	REPgwR	ATCTTCTATTTCAACACCACCATCGTTTTCC TC	
23	BP-rep-F	GGGGACAAGTTTGTACAAAAAAGCAGGCT TCATGATCGACATCCCTCCTTAC	cloning *REPΔC*
24	BP-rep-R	GGGG ACCACTTTGTACAAGAA AGCTGGGTCGAGCTTTTCATATAGCTTCAC	cloning *REPΔC*
25	REP-F3	GACCAAGGGATAAAGGCGCT	qRT-PCR
26	REP-R4	ACCTCCAAATTCACCCTTGACT	qRT-PCR
27	REP-F5	TGGAATCAGCCGTGAAGCTATATG	qRT-PCR
28	REP-R6	TCGTTTTCCTCCTCCGTTGTG	qRT-PCR
29	g3GFP-R	CCATCTAATTCAACAAGAATTGGGACAAC	RT-PCR of revertant
30	PP2a-F	TAACGTGGCCAAAATGATGC	RT-PCR control gene
31	PP2A-R	GTTCTCCACAACCGCTTGGT	RT-PCR control gene
